# HSP90AB1‐Mediated Ubiquitin‐Proteasome Degradation of ITGBL1 Promotes Osteosarcoma Progression by Inhibiting Endoplasmic Reticulum Stress‐Induced Autophagy

**DOI:** 10.1002/advs.202515651

**Published:** 2026-02-16

**Authors:** Zhen Wang, Zixuan Guo, Chengwei Cao, Ziying Wang, Zifu Huang, Xiujuan Zhang, Yushu Zheng, Diankun She, Hao Zhu, Lingfeng Yu, Xuelin Zhao, Dongquan Xiang, Song Liao, Xin He, Xintong Ji, Chengsheng Wu, Cheng‐Xiong Xu, Meng Xu

**Affiliations:** ^1^ Medical School of Chinese PLA Beijing China; ^2^ Senior Department of Orthopedics the Fourth Medical Centre Chinese PLA General Hospital Beijing China; ^3^ Department of Cell Engineering Beijing Institute of Biotechnology Beijing China; ^4^ Department of Orthopedics Jinling Hospital Affiliated Hospital of Medical School Nanjing University Nanjing Jiangsu China; ^5^ Institute of Basic Medical Sciences Chinese Academy of Medical Sciences School of Basic Medicine Peking Union Medical College Beijing China; ^6^ Department of Orthopedic Oncology Shanghai Bone Tumor Institute Shanghai General Hospital Shanghai Jiao Tong University School of Medicine Shanghai China; ^7^ College of Life Sciences University of Chinese Academy of Sciences Beijing China; ^8^ School of Medicine Chongqing University Chongqing China

**Keywords:** Autophagy, ER stress, HSP90AB1, ITGBL1, K63‐linked ubiquitination, Osteosarcoma

## Abstract

Osteosarcoma (OS) is one of the most malignant bone tumors in children and adolescents, but the molecular mechanisms of OS progression remain largely undefined. In this study, we demonstrate that Integrin subunit beta‐like 1 (ITGBL1) is downregulated in OS tissues, and its downregulation correlates with poor prognosis in OS patients. Functional assays revealed that ITGBL1 inhibits OS cell growth, metastasis, and stemness, while promoting apoptosis. The in vitro and in vivo experiments further revealed that ITGBL1 activates endoplasmic reticulum (ER) stress by upregulating ROS, thereby triggering autophagy in OS cells. In addition, the downregulation of ITGBL1 in OS is partly attributed to the abnormal upregulation of HSP90AB1 (heat shock protein 90 alpha family class B1). Mechanistically, ITGBL1 interacts with HSP90AB1 which facilitates ITGBL1 degradation through K63‐linked ubiquitination. Finally, through virtual screening and Co‐IP, we identified ivermectin as a potent inhibitor of the HSP90AB1‐ITGBL1 interaction, and treatment with ivermectin dramatically inhibited OS progression in vivo. In conclusion, we uncover a novel mechanism that promotes OS progression and identify a new candidate drug for the treatment of OS.

## Graphical abstract

This study delineates a novel HSP90AB1‐ITGBL1 signaling axis governing osteosarcoma. HSP90AB1 promotes K63‐linked ubiquitination and proteasomal degradation of ITGBL1. Restoring ITGBL1 induces reactive oxygen species‐dependent endoplasmic reticulum stress and pro‐death autophagy, suppressing tumor growth and metastasis. The drug ivermectin disrupts this interaction, offering a repositioning strategy for targeted therapy.

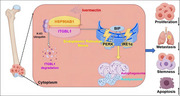

## 1 Introduction

Osteosarcoma (OS) is the most prevalent primary malignant bone tumor in children and adolescents, with an annual incidence of 3–4.5 cases per million people worldwide [1]. Multimodal management (radical surgery combined with cisplatin‐, doxorubicin‐, and high‐dose methotrexate‐based neoadjuvant chemotherapy) has increased the 5‐year overall survival rate of localized OS patients to approximately 60% [2]. However, the 5‐year survival for patients with metastatic or relapsed OS is less than 20%, and there is currently no effective treatment method or targeted drugs [3]. Thus, to develop new therapeutic strategies or new targeted drugs that can effectively inhibit the progression of OS, we first need to elucidate the molecular mechanisms that promote OS progression.

The Integrin subunit beta‐like 1 (ITGBL1), initially cloned from an osteoblast cDNA library, encodes a β‐integrin‐related extracellular matrix protein [4]. ITGBL1 is highly homologous to the N‐terminal EGF‐like stalk fragment of integrin β, but contains neither a transmembrane domain nor an RGD (Arg–Gly–Asp)‐binding domain, suggesting that ITGBL1 functions distinctly from those of integrins [5]. Recent evidence indicates that ITGBL1 is abnormally expressed in various cancers and plays an important role in the metastasis of various cancers [6, 7, 8], including hepatocellular carcinoma as well as breast, ovarian, and gastric cancers [5, 6, 8, 9]. Notably, ITGBL1 exhibits context‐dependent and even opposing roles across cancer types. For instance, ITGBL1 is downregulated in non‐small cell lung cancer (NSCLC), and its downregulation promotes the progression of NSCLC by activating Wnt/PCP signaling [10]. In contrast, high expression of ITGBL1 in breast cancer promotes bone metastasis by activating TGF‐β signaling [5]. However, the expression profile and functional significance of ITGBL1 in OS, as well as the mechanisms governing its dysregulation in cancer, remain to be elucidated.

Heat shock protein 90 alpha family class B member 1 (HSP90AB1), a core member of the HSP90 family that functions as a molecular chaperone, playing a pivotal role in maintaining cellular protein homeostasis and regulating diverse biological processes [11]. The HSP90AB1 gene, which encodes the HSP90B protein, contributes to oncogenesis and other pathologies by coordinating the maturation and stabilization of a broad repertoire of client proteins [12, 13]. For example, HSP90AB1 stabilizes LRP5 to promote metastasis by activating the AKT and Wnt/β‐catenin signaling pathways in gastric cancer [14]. In lung adenocarcinoma, HSP90AB1 promotes metastasis by activating TGF‐β/SMAD signaling through interactions with EEF1A2 [15]. In OS, single‐cell RNA‐sequencing data suggest that HSP90AB1 may be involved in the regulation of OS metastasis [16], however, the precise expression patterns, biological functions, and mechanistic contributions of HSP90AB1 in OS have yet to be fully defined.

The endoplasmic reticulum (ER) is a central organelle in which transmembrane proteins are synthesized, folded, and modified [17], while energy stress, oxidative stress, nutrient deprivation, and dysregulated calcium levels can induce unfolded protein response (UPR) or ER stress response [18]. Cells can rapidly respond to ER dysfunction by activating ER stress sensors such as transcription factor 6 (ATF6), inositol‐requiring enzyme 1α (IRE1α), and PRKR‐like ER kinase (PERK). The molecular chaperone binding‐immunoglobulin protein (BIP; also known as GRP78) is an inhibitor of ER stress sensors, which inhibits the ER stress response by binding to sensors [19]. During the period of ER stress, BIP shows increased binding affinity for misfolded or unfolded proteins and therefore dissociates from ER stress sensors, which is considered the initial period of ER stress [20]. Depending on the magnitude of ER stress, the cell type, and the specific pathological context, ER stress responses can have multiple effects ranging from cellular reprogramming and adaptation to autophagy and apoptosis [21]. Autophagy is a process of degradation of intracellular components, including cytoplasmic contents, (part of) organelles, membranes, proteins, and nucleic acids, known as autophagosomes. Then, the contents of the autophagosome are transferred to lysosomes for degradation [22]. ER stress‐induced autophagy can function as a double‐edged sword in cancer, as it can either promote or restrict tumor growth depending on the disease type and genetic environment [23, 24].

In this study, we first demonstrated that ITGBL1 is expressed at low levels and its downregulation predicts poor prognosis in OS patients. ITGBL1 overexpression activated ER stress through upregulating ROS in OS cells, which induced autophagy, thereby preventing OS progression. Furthermore, Co‐IP and Mass Spectrometry (MS) revealed that HSP90AB1 directly interacts with ITGBL1 and promotes the degradation of ITGBL1 protein through the K63‐linked ubiquitination pathway. Finally, we identified ivermectin as a small‐molecule inhibitor that disrupts the binding of HSP90AB1 and ITGBL1, exerting a potent suppressive effect on OS progression in vivo. Collectively, these results elucidate a novel regulatory axis and propose a promising therapeutic candidate for OS treatment.

## 2 Materials and Methods

### 2.1 Sample Collection

OS tissues and paired normal bone tissues were obtained from 18 OS patients who were admitted in Chinese PLA General Hospital (Beijing, China). The research protocol was approved by the Ethics Committee of Chinese PLA General Hospital (2023KY032‐HS001). All participating patients provided written informed consent authorizing the use of specimens for the intended research. All resected specimens were stored at −80°C.

### 2.2 Cell Culture

MG63, U_2_OS, HOS, 143B, hFOB1.19, and 293T cells were obtained from the Cell Bank of the Chinese Academy of Sciences (Shanghai, China). 143B cells were maintained in Eagle's minimum essential medium (EMEM) supplemented with 10% FBS (Gibco, Thermo Fisher Scientific). Other cell lines were cultured in DMEM (Gibco, Thermo Fisher Scientific) supplemented with 10% fetal bovine serum (FBS) (Gibco, Thermo Fisher Scientific). All cells were incubated in 5% CO_2_ at 37°C in a humidified atmosphere.

### 2.3 Cell Transfection

FLAG‐ITGBL1, GFP‐ITGBL1, HA‐HSP90AB1, HIS‐ubiquitin, FLAG‐tagged ITGBL1‐truncations (ITGBL1 amino acids 142–494, △142–229, △230–366, and 1–366) and HA‐tagged HSP90AB1‐truncations (HSP90AB1 amino acids 1–620, 233–724, △233–620) were purchased from Miaoling Biotechnology (Wuhan, China). The short hairpin RNAs (shRNAs) targeting ITGBL1 and HSP90AB1 were purchased from GenePharma (Shanghai, China). OS cells were transfected with the indicated plasmids using Lipofectamine 2000 reagent (Invitrogen, CA, USA) according to the manufacturer's protocol. To establish stable cell lines, the lentivirus‐containing plasmids infected 143B cell line. Then, 48 h after infection, cells were selected with puromycin(2 µg/mL) for 2 weeks to construct a stable cell line. The sequences of shRNAs are listed in Table S1.

### 2.4 Immunohistochemistry (IHC)

For IHC analysis, the slides were deparaffinized with xylene, rehydrated with ethanol, and incubated with 3% H_2_O_2_ for 5 min to block endogenous peroxidase activity. Then, antigen retrieval was performed by incubating the samples with sodium citrate buffer (pH 6.0) for 20 min at 95°C. After blocking with 5% normal goat serum for 10 min at 20°C, the sections were incubated with primary antibodies at 4°C overnight and then incubated with secondary antibodies (1:200, Servicebio, China). The images were captured with the Olympus FSX100 microscope (Olympus, Japan). The average optical density in the tumor was calculated using ImageJ and reviewed by two pathologists. ITGBL1 primary antibody was purchased from Abcepta (1:200, China), Ki67 primary antibody was purchased from Servicebio (1:500, China), and antibodies against HSP90AB1 (1:250), OCT4 (1:200), NANOG (1:200), and SOX2 (1:200) were purchased from Proteintech (China).

### 2.5 RNA Extraction and RT‐qPCR

Total RNA was extracted from cells and tissues using Trizol Reagent (Invitrogen, CA, USA) according to the manufacturer's instructions. For the quantification of mRNA, 1 µg of total RNA was reverse‐transcribed to cDNA using HiScript III first Strand cDNA Synthesis Kit (Vazyme, Nanjing, China). RT‐qPCR was performed using SYBR Green dye (Vazyme) with LightCycler96 System (Roche, IN, USA). All reactions were run in triplicate. After the reactions were complete, the cycle threshold (*CT*) values were determined with the LightCycler96 software. A comparative CT method was used to compare each condition to the control reactions. GAPDH was used as an internal control, and the relative level was calculated with the equation 2^−ΔΔ^ *^CT^*. The primer sequences of ITGBL1, HSP90AB1, BIP, and GAPDH are listed in Table S2.

### 2.6 Immunofluorescence (IF)

OS cells were washed with 1 × PBS and fixed with 4% paraformaldehyde at room temperature for 30 min. Fixed cells were blocked with PBS containing 5% BSA (Servicebio) for 30 min, followed by permeabilization with 0.1% Triton X‐100 (Servicebio) in PBS for 10 min. Then incubate the cells overnight at 4°C with the primary antibody diluted with a blocking solution. After three washes with PBS, cells were stained with Alexa Fluor 488 (Abcam, USA)‐ or Alexa Fluor 594 (Abcam, USA)‐conjugated secondary antibodies at room temperature for 1 h in the dark. DAPI was used to counterstain nuclei for 10 min. Images were visualized by a confocal microscope (Nikon, Japan). Subsequently, target regions were accurately delineated in each image to measure their mean fluorescence intensity (Mean = IntDen/Area) with Image J (NIH, USA). The background value, obtained from a cell‐free area within the same field of view, was then subtracted to yield the net fluorescence intensity. Finally, data from all fields of view per sample were aggregated and subjected to statistical inter‐group comparisons, thereby achieving objective quantification of protein expression levels [25]. All primary antibodies used for IF were purchased from Proteintech. The antibody dilution ratio was 1:200.

### 2.7 Cell Viability Assay

The proliferation capability of OS cells was assessed by cell counting kit‐8 assay (CCK‐8) according to the manufacturer's instructions (Yeasen, Shanghai, China). The indicated cells were seeded into 96‐well plates (5 × 10^3^ cells/well). At 12, 24, 36, and 48 h after seeding or drug treatment, 10 µL of CCK‐8 reagent was added to the test well and incubated for 2 h at 37°C away from light. The absorbance was measured at a wavelength of 450 nm using a microplate reader (Thermo Scientific, CA, USA).

### 2.8 Wound Healing and Transwell Assay

Wound healing assays were performed to evaluate the migratory ability of OS cells. After 24‐h transfection, 143B and HOS cells were seeded into six‐well plates (6 × 10^5^ cells/well). After 24‐h seeding, pipette tips (200 µL) were used to scrape a straight scratch in the confluent cell layer and then cultured in 2% fetal bovine serum (FBS) medium. After washing the cells with PBS to remove cellular fragments, each wound was imaged at 0 and 24 h under the microscope at 100×. The diminishing area among scratch wound was analyzed by Image J in 24 h, and normalized to the scratched area in 0 h.

Invasion and migration assays were conducted in 24‐well plates using a Transwell chamber (Corning, NY, USA) with and without Matrigel (BD Science, MA, USA) coating, respectively. After 24‐h of transfection, 5 × 10^4^ cells for migration and 1 × 10^5^ cells for invasion were suspended in 200 µL serum‐free DMEM medium and seeded into the upper of the Transwell chamber. DMEM medium (500 µL) containing 20% FBS was then filled into the bottom of the Transwell chamber. After 24‐h culture, the filters were fixed in methanol and stained with 0.1% crystal violet. The upper cells of the filters were gently abraded, and the lower cells that migrated across the filters were imaged and counted. The number of migrated cells was counted and calculated by Image J.

### 2.9 Sphere Formation Assay

To evaluate sphere formation efficiency, approximately 100 cells per well were seeded into an ultra‐low attachment 96‐well plate (Corning). Cells were cultured in serum‐free DMEM/F12 medium (Gibco) supplemented with epidermal growth factor (EGF, 20 ng/mL; Gibco), basic fibroblast growth factor (bFGF, 20 ng/mL; Gibco), 1 × B27 (Invitrogen), and 2% methylcellulose (Sigma‐Aldrich). The sphere formation was assessed 7 days after seeding. The spheres were stained with Calcein‐AM, and imaged under a microscope (Olympus). The sphere diameter was measured using Image J [26, 27, 28].

### 2.10 Flow Cytometry Analysis

After 48‐h of transfection, cells were digested, centrifuged, and washed twice with cold PBS. The cells were incubated in the dark with APC‐labelled CD133 flow cytometry antibody (BioLegend, CA, USA) for 20 min at room temperature. Then, the cells were washed and resuspended with PBS and detected CD133 positive cells by flow cytometry. The data were analyzed with FlowJo v10.7.1 software (BD Science).

### 2.11 TUNEL Assay

Cell apoptosis was detected using the TUNEL Apoptosis Detection Kit (Yeasen) following the manufacturer's instructions. The OS cells were fixed on the slide with 4% acetaldehyde for 15 min, and washed. Then incubated with 0.5% Triton X‐100 solution for 10 min. Then washing by phosphate buffered saline, the cells were incubated in the dark with TUNEL reagent at 37°C for 60 min. After staining, the cells were washed and then incubated with DAPI for 10 mins. Cell apoptosis detection was performed with a fluorescence microscope.

### 2.12 Co‐immunoprecipitation (Co‐IP)

Co‐IP was performed as described previously [29]. Briefly, OS cells were transfected with the indicated plasmids for 48‐h, then the cells were harvested and lysed. The cell lysates were incubated with Protein A/G magnetic beads (MCE, USA) coated with specified antibodies or isotype IgG at 4°C for overnight with rotation. The next day, after washing the beads three times with inhibitor lysate, 50 µL of SDS‐PAGE Sample Loading Buffer (1×) was added. The mixtures were subsequently heated for 5 min at 95°C, and the beads were isolated by incubation for 10 s on a magnetic rack. The supernatant was then used for Western blotting.

### 2.13 Western Blot

The proteins were separated in SDS‐polyacryl‐amide gel electrophoresis (SDS‐PAGE) and transferred to 0.22 µm PVDF membranes (Millipore, Massachusetts, USA). The membrane was sealed with 5% skim milk and then incubated with primary antibodies at 4°C overnight. Then, the membrane was washed with 1 × TBST buffer for 10 min × 3. The membrane was incubated with secondary antibodies for 1 h at room temperature, and washed with 1 × TBST buffer for 10 min × 3. Then, the membrane was incubated with ECL substrate (Thermo Fisher, CA, USA) according to the manufacturer's instructions, and the bands were detected with ChemiDoc Imaging Systems (Bio‐Rad, USA). The protein bands were analyzed with ImageJ (NIH).

### 2.14 Ubiquitination Assay

To detect the ubiquitination of endogenous ITGBL1, OS cells were treated with 20 µm MG132 (MCE, USA) for 9 h to inhibit proteasome‐mediated degradation. Then, ITGBL1 was immunoprecipitated using the indicated antibodies and subjected to immunoblotting. For the analysis of ITGBL1 protein half‐life, cells were treated with 100 µg/mL cycloheximide (MCE, USA) for 0, 2, 4, and 8 h. At each time point, cells were harvested, and ITGBL1 protein levels were assessed by immunoblotting. The relative protein levels were quantified by densitometry analysis to determine the degradation rate over time.

### 2.15 Autophagy Assay

Cells were monitored for autophagic flux using a tandem LC3B tagged with mCherry and GFP. MCherry‐GFP‐LC3B (Miaoling Biotechnology) and ITGBL1 overexpression plasmids transfected 143B and HOS cells (1 × 10^4^ cells) were seeded in confocal dishes and incubated overnight. The next day, the culture media were discarded, and the cells were washed three times with PBS. To assess autophagy flux, both GFP and mCherry LC3B dots were analyzed to distinguish autophagosomes (GFP^+^ mCherry^+^) from autolysosomes (mCherry + only). Images were acquired with a confocal microscope (Nikon, Japan) [30].

### 2.16 Transmission Electron Microscopy (TEM)

Transmission electron microscopy (TEM) was applied to analyze the ultrastructural changes of the endoplasmic reticulum and the autophagosomes or autolysosomes formation in cells. After indicated treatment, 143B and HOS cells were washed with PBS and collected in a 1.5 mL EP tube. Cells were pre‐fixed with 2.5% glutaraldehyde and 4% paraformaldehyde at 4°C for 2 h, washed with 0.1 m phosphate buffer, and then post‐fixed with 1% osmium acid at 4°C for 2 h before being dehydrated with gradient concentrations of acetone. The samples were subsequently embedded, polymerized, and sectioned. Ultrathin sections were stained with uranyl acetate and lead citrate for 15 min and examined by a HITACHI H‐7650 transmission electron microscope.

### 2.17 Bioinformatic Analysis

The gene expression and clinical data of OS patients were collected from the TARGET (https://ocg.cancer.gov/programs/target) database. The gene expression profiles from GSE33382 (including 84 OS samples and 3 normal osteoblast samples; platform: GPL10295) and GSE19276 (including 43 OS samples and 4 normal bone samples; platform: GPL6848) were obtained from the GEO database (http://www.ncbi.nih.gov/geo).

### 2.18 Molecular Docking

Rigid protein–protein docking between HSP90AB1 and ITGBL1 was performed using GRAMM‐X to investigate their interactions. Protein structures were sourced from the UniProt database (www.uniprot.org), the PDB database (RCSB PDB: Homepage), and the AlphaFold database. PyMOL (Version 2.4) was utilized for the analysis of protein interactions and subsequent visualization.

### 2.19 Virtual Screening

Molecular docking studies were performed using the semi‐flexible docking algorithm of AutoDock Vina (version 1.1.2) and were compiled and run under the Windows 10 operating system. The crystal structure of ITGBL1 was defined as a receptor. The 2115 compounds from the ZINC database were defined as the virtual screening library. All compounds were prepared using OpenBabel. The docking poses were scored using the Vina scoring function, which considers various intermolecular interactions like van der Waals forces and hydrogen bonds. Up to 10 conformations of each ligand were considered in the docking process, and we selected the top five compounds with the lowest binding energies as candidates for further molecular dynamics analysis via GROMACS. All structural reconstructions were produced using Pymol.

### 2.20 Animal Experiments

Male BALB/c nude mice (4 weeks old) were purchased from Gempharmatech Co., Ltd. (Nanjing, China), and randomly divided into different groups (*n* = 5/group). For the tumorigenicity assay, mice were randomly divided into three groups and subcutaneously injected with 143B cells mixed with Matrigel (Corning, USA). Each mouse received ITGBL1 shRNA‐transfected cells on the right flank and scramble control cells on the left flank at the following doses: 5 × 10^5^, 1 × 10^5^, or 5 × 10^4 ^cells per site. Mice were euthanized 6 weeks after injection. The frequency of cancer stem cells (CSCs) was then calculated using extreme limiting dilution analysis (ELDA) (https://bioinf.wehi.edu.au/software/elda/) [31]. To construct an orthotopic OS model, luciferase‐labelled 143B cells transfected with the indicated plasmids were inoculated into the tibia of nude mice (1 × 10^7^ cells/mouse). The tumor growth was monitored with a Xenogen IVIS Spectrum Imaging System (PerkinElmer, USA), and the tumor volume was measured every week after implantation. The tumor volume was calculated according to the formula: tumor volume [mm^3^] = (length [mm]) × (width [mm])^2^ × 0.52. The mice were sacrificed after 28 days, and tumors were excised and weighed. Each tumor was divided into two parts, one for Co‐IP analysis and the other fixed in 4% paraformaldehyde for hematoxylin and eosin (H&E) staining, IHC staining, and TUNEL staining. To construct an OS lung metastasis model, luciferase‐labelled 143B cells (1 × 10^5^ cells/mouse) were injected into the tail veins of mice. OS cell lung metastasis was monitored with a Xenogen IVIS Spectrum Imaging System. After 4 weeks, the lungs of mice were excised under anesthesia, and the number of lung metastatic nodules was counted and processed with HE staining, immunohistochemical staining, and TUNEL staining as well. All animal experiments were approved by the Institutional Animal Care and Use Committee of the Chinese PLA General Hospital (S2013‐115‐01).

### 2.21 Statistical Analysis

All data were expressed as the mean ± standard deviation (SD). Statistical analyses were performed using Prism software (GraphPad Software 8), and consisted of analysis of variance followed by Student's *t*‐test when comparing two experimental groups. One‐way ANOVA analysis was used to compare the differences between groups. Overall survival (OS) and progression‐free survival (PFS) were determined with the Kaplan–Meier method. All experiments were triplicated, and *p* < 0.05 was considered statistically significant.

## 3 Results

### 3.1 ITGBL1 Is Downregulated in OS, and Low Expression of ITGBL1 Is Related to Poor Prognosis in OS Patients

**FIGURE 1:**
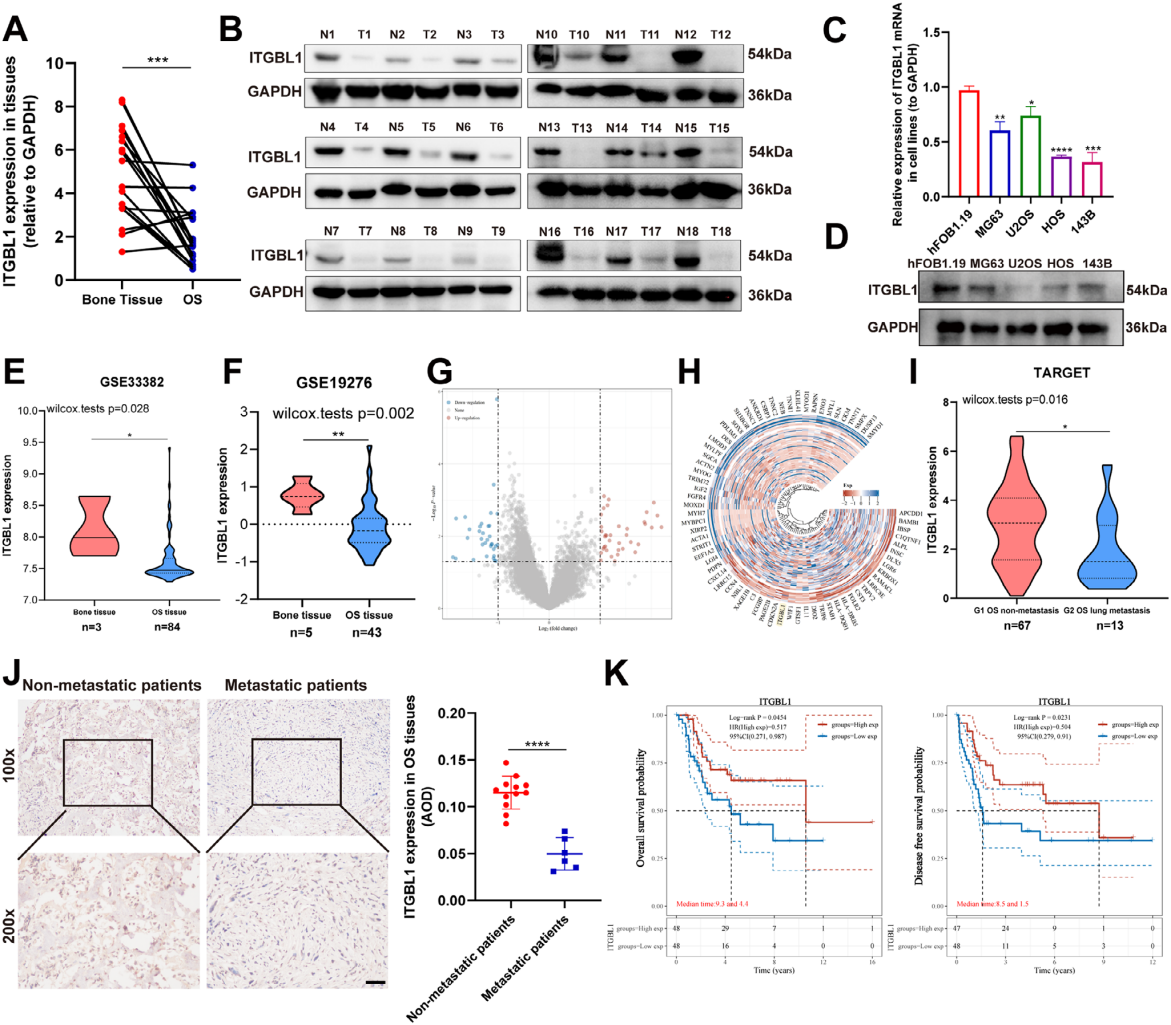
ITGBL1 is downregulated in OS, and low expression of ITGBL1 is related to poor prognosis in OS patients. (A,B) The mRNA and protein expression levels of ITGBL1 in OS tissues and their normal bone tissues (*n* = 18 pairs). (C,D) The mRNA and protein expression levels of ITGBL1 in the indicated cell lines. (E) The mRNA expression level of ITGBL1 in normal osteoblast and OS tissues. Data from GSE33382. (F) The mRNA expression level of ITGBL1 in bone tissues and OS tissues. Data from GSE19276. (G,H) The volcano map and heatmap show the differentially expressed genes between primary tumors of with or without lung metastasis (log*FC* > 2 and *P.adj* < 0.05). Data from the TARGET database. (I) The mRNA expression level of ITGBL1 in primary tumors of OS patients with or without lung metastasis. Data from the TARGET database. (J) IHC staining of ITGBL1 in primary tumor tissues of OS patients with (*n* = 12) and without lung metastasis (*n* = 6). Scale bar = 50 µm. (K) Kaplan–Meier analyses of the overall survival rate and disease‐free survival rate of OS patients with high and low expression of ITGBL1. All in vitro experiments were conducted three independent experiments. All data are presented as the mean ± SD, and differences between groups were assessed by Student's *t*‐test. * *p* < 0.05; ** *p* < 0.01; *** *p* < 0.001; **** *p *< 0.0001.

First, we investigated whether the expression of ITGBL1 is abnormal in OS. The analysis of clinical samples revealed that ITGBL1 is significantly downregulated in human OS tissues compared to normal bone tissues at both the mRNA (Figure 1A) and protein levels (Figure 1B). Consistently, we demonstrated lower expression of ITGBL1 in OS cell lines than in osteoblast hFOB1.19 cells at both the mRNA (Figure 1C) and protein levels (Figure 1D). These results were further supported by the Gene Expression Omnibus (GEO) datasets GSE33382 and GSE19276. As shown in Figure 1E,F, ITGBL1 mRNA was significantly downregulated in OS samples compared with normal bone tissues. Importantly, analysis of RNA‐sequencing data from the therapeutically applicable research to generate effective treatments (TARGET) cohort revealed that transcript levels of ITGBL1 were significantly lower in primary OS tissues from lung metastasis patients (*n* = 13) than in those from nonmetastatic patients (*n* = 67) (Figure 1G–I). This result was further confirmed using our clinical sample cohort by immunohistochemical (IHC) analysis (Figure 1J). Notably, TARGET cohort analysis revealed that low expression of ITGBL1 was significantly correlated with a lower overall survival (OS) rate and a lower disease‐free survival (DFS) rate (Figure 1K) in OS patients. Together, these results suggest that ITGBL1 is expressed at low levels in OS patients and that low expression of ITGBL1 is closely related to poor prognosis in OS patients.

### 3.2 Downregulation of ITGBL1 Promotes OS Progression and Stemness, but Inhibits the Apoptosis of OS Cells

**FIGURE 2:**
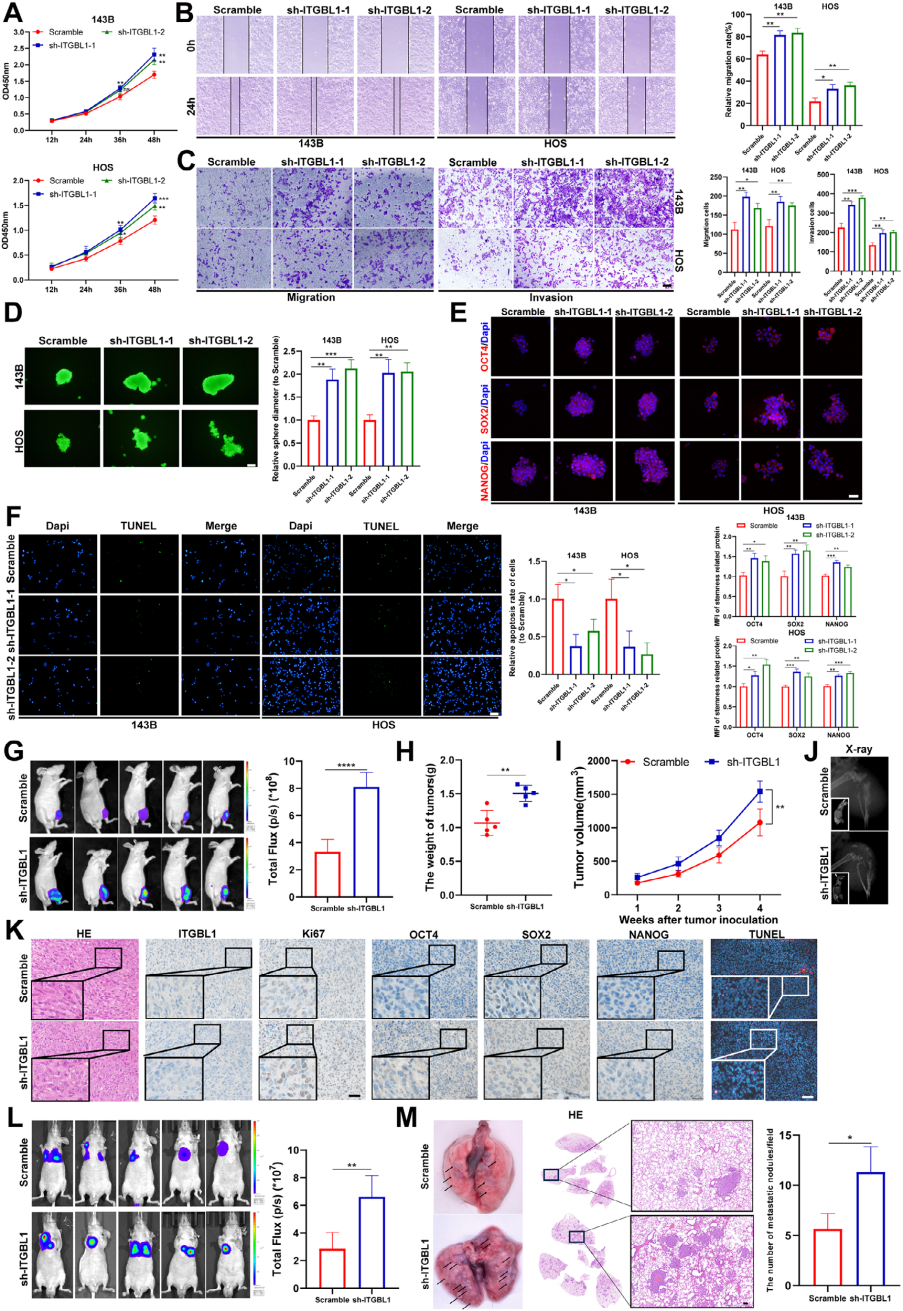
Downregulation of ITGBL1 promotes OS progression and stemness, while inhibiting apoptosis of OS cells. (A) OS cell viability was measured by the CCK8 assay. (B) OS cell migration was measured by the wound healing assay. Scale bar = 100 µm. (C) OS cell migration and invasion were measured by the Transwell assay. Scale bar = 100 µm. (D) The stemness of OS cells was measured by sphere formation assay, and the spheres were stained with Calcein‐AM and imaged. Scale bar = 50 µm. (E) The expression of stemness‐related proteins was detected by immunofluorescence in spheres. Scale bar = 50 µm. (F) The apoptotic cells were detected in 143B and HOS cells by TUNEL assay. Scale bar = 100 µm. (G) Bioluminescence images and quantification of orthotopic OS models (*n *= 5/group). (H,I) The tumor weight and growth in OS orthotopic models. (J) Typical X‐ray images of the tibia in the orthotopic OS model. (K) HE, IHC (ITGBL1, Ki67, OCT4, SOX2, and NANOG), and TUNEL assays of orthotopic xenograft tumors. Scale bar = 50 µm in images of HE and IHC; Scale bar = 100 µm in TUNEL images. (L) Bioluminescence images and quantification of OS lung metastasis models. (M) Representative images of Lung and lung tissue HE staining in OS lung metastasis models. Scale bar = 100 µm. All in vitro experiments were conducted in three independent experiments. All data are presented as the means ± SD, and differences between groups were assessed by Student's *t*‐test. * *p* < 0.05; ** *p* < 0.01; *** *p* < 0.001; **** *p* < 0.0001.

To investigate whether the downregulation of ITGBL1 is directly involved in the progression of OS, we conducted functional experiments using OS cells with ITGBL1 knockdown (Figure S1A,B). CCK‐8 assay revealed that knockdown of ITGBL1 significantly promoted OS cell proliferation (Figure 2A), while wound healing (Figure 2B), migration, and invasion (Figure 2C) assays revealed that knockdown of ITGBL1 significantly enhanced the metastasis of OS cells. The knockdown of ITGBL1 also enhanced the sphere formation ability of OS cells (Figure 2D), upregulated the expression of stemness‐related proteins such as OCT4, SOX2, and NANOG (Figure 2E), and increased the proportion of CD133‐positive cells in OS cells [32] (Figure S2A). Then, we investigated the effect of ITGBL1 downregulation on OS cell apoptosis by the TUNEL assay. Our results showed that knockdown of ITGBL1 significantly inhibited apoptosis in both 143B and HOS cells compared to the scramble control group (Figure 2F). These in vitro experimental results were further confirmed by animal models. As shown in Figure S2B, that knockdown of ITGBL1 significantly enhanced the tumorigenic ability of 143B cells in nude mice. The fluorescence intensity (Figure 2G), tumor weight (Figure 2H), and tumor volume (Figure 2I) data of orthotopic OS models showed that 143B cells with stable ITGBL1 knockdown exhibited significant growth advantages in nude mice compared to control 143B cells. In addition, the X‐ray image showed that knockdown of ITGBL1 exacerbated OS cell‐induced bone destruction (Figure 2J). We further analyzed the expression levels of cell proliferation and stemness marker proteins, as well as apoptosis in tumors harvested from the orthotopic OS model. The expression of Ki67, OCT4, SOX2, and NANOG was markedly elevated in ITGBL1‐knockdown tumors, which also exhibited fewer apoptotic cells compared with control tumors (Figure 2K). To investigate whether ITGBL1 is involved in OS metastasis in vivo, we established an OS lung metastasis model by tail vein injection of luciferase‐labeled 143B cells. Bioluminescence imaging revealed greater lung fluorescence intensity in the ITGBL1‐knockdown group compared with the scramble control group (Figure 2L). Consistently, lung images and lung tissue HE analysis at the experimental endpoint demonstrated more than a twofold increase in the number of lung metastatic nodules in the ITGBL1‐knockdown group (Figure 2M). Taken together, these data suggest that downregulation of ITGBL1 strongly contributes to OS progression by facilitating OS cell proliferation, metastasis, stemness, and inhibiting apoptosis.

### 3.3 Overexpression of ITGBL1 Inhibits OS Progression

**FIGURE 3:**
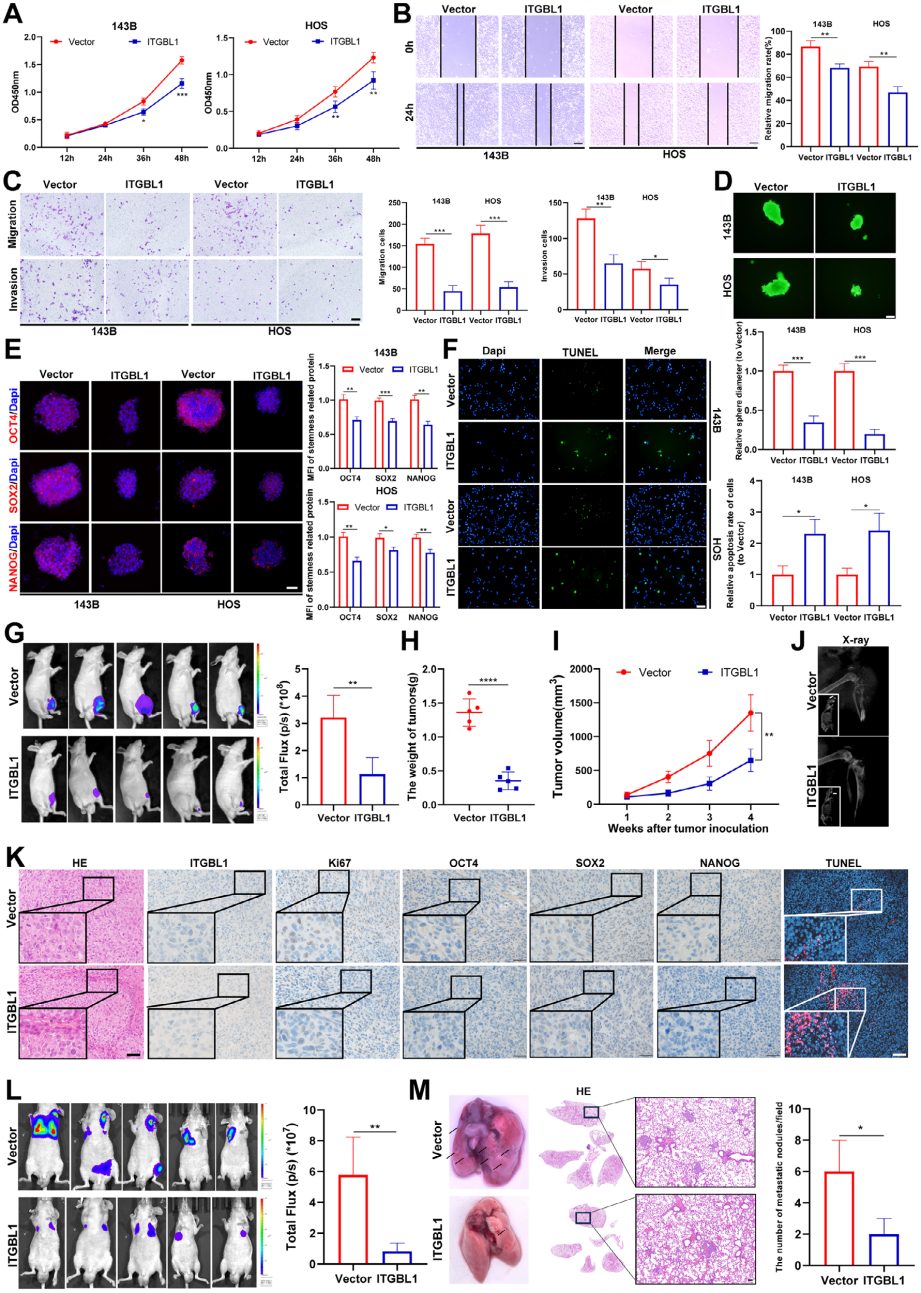
Overexpression of ITGBL1 inhibits OS progression. (A) OS cell viability was measured by the CCK8 assay. (B) OS cell migration was measured by the wound healing assay. Scale bar = 100 µm. (C) OS cell migration and invasion were measured by the transwell assay. (D) OS cell stemness was measured by sphere formation assay. Scale bar = 50 µm. (E) The expression of stemness‐related proteins was detected by immunofluorescence in OS spheres. Scale bar = 50 µm. (F) Apoptotic cells in OS cells were detected by the TUNEL assay. Scale bar = 100 µm. (G) Bioluminescence images and quantification of orthotopic OS model (*n *= 5). (H,I) The tumor weight and growth in the orthotopic OS model. (J) Typical X‐ray image of tibia in the orthotopic OS model. (K) HE, IHC (ITGBL1, Ki67, OCT4, SOX2, and NANOG), and TUNEL staining of orthotopic xenograft tumors. Scale bar = 50 µm in images of HE and IHC, Scale bar = 100 µm in TUNEL images. (L) Bioluminescence images and quantification of OS lung metastatic models. (M) Representative images of Lung and lung tissue HE staining in OS lung metastasis models. Scale bar = 100 µm. All in vitro experiments were conducted three independent experiments. All data are presented as the means ± SD, and differences between groups were assessed by Student's *t*‐test. * *p* < 0.05; ** *p* < 0.01; *** *p* < 0.001; **** *p* < 0.0001.

The results above indicate that the downregulation of ITGBL1 promotes OS progression, suggesting that the overexpression of ITGBL1 may inhibit OS progression. As expected, in vitro experiments revealed that the overexpression of ITGBL1 (Figure S1C,D) significantly suppressed OS cell proliferation (Figure 3A), migration, and invasion (Figure 3B,C). In addition, the overexpression of ITGBL1 significantly inhibited OS cell sphere formation (Figure 3D) and the expressions of OCT4, SOX2, and NANOG in OS spheres (Figure 3E), but the overexpression of ITGBL1 promoted the apoptosis of OS cells (Figure 3F). Similar results were observed in vivo. The orthotopic xenograft model experiment showed that overexpression of ITGBL1 significantly inhibited tumor growth (Figure 3G,I), OS cell‐induced bone destruction (Figure 3J), and the expression of Ki67, OCT4, SOX2, and NANOG in tumors (Figure 3K). In addition, the overexpression of ITGBL1 significantly suppressed OS cell lung metastasis (Figure 3L,M). These results indicate that overexpression of ITGBL1 can inhibit OS progression.

### 3.4 ITGBL1 Activates ER Stress and Autophagy in OS Cells Through Upregulating ROS

**FIGURE 4:**
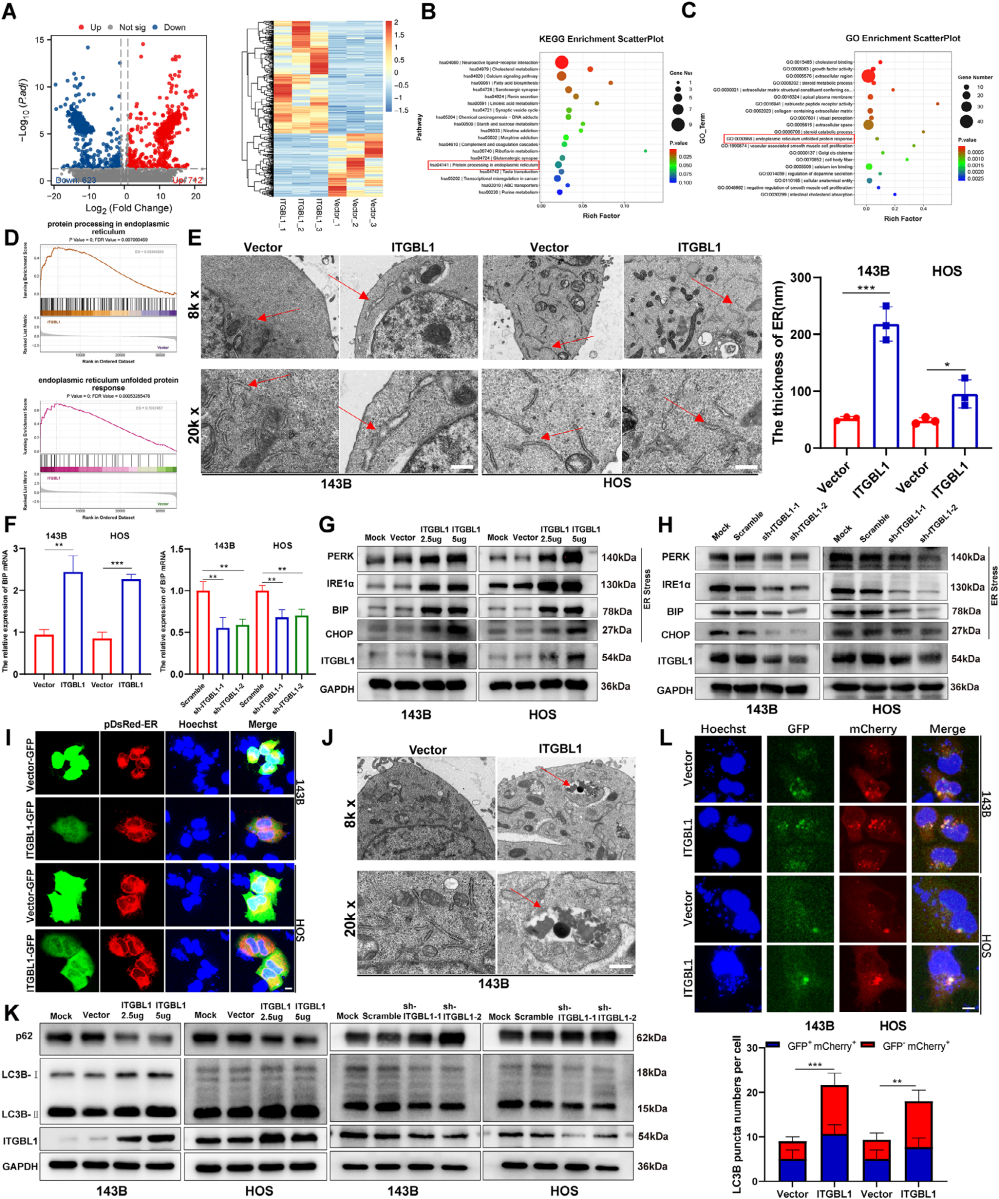
ITGBL1 activates ER stress and autophagy in OS cells. (A) Differentially expressed genes between ITGBL1 overexpression and the vector group with log_2_ *FC* ≥ 1 and *P.adj* < 0.05 were presented with a volcano map and a heatmap. (B,C) KEGG and GO pathway enrichment analysis of DEGs. (D) GSEA shows that ITGBL1 expression levels are positively correlated with the response to endoplasmic reticulum (ER) stress and endoplasmic reticulum unfolded protein response. (E) The morphology and thickness changes of ER in OS cells were examined using transmission electron microscopy (TEM), and the ER was indicated by a red arrow. Scale bar = 500 nm. (F) The expression level of BIP mRNA was detected by RT‐qPCR. (G,H) The expression of ER stress‐related proteins including PERK, IRE1α, BIP, and CHOP were detected by Western blot. (I) ITGBL1‐GFP or Vector‐GFP and pDsRed‐ER plasmids were transfected into 143B and HOS cell lines for confocal microscopic analysis. Scale bar = 10 µm. (J) Autolysosomes in 143B cells were detected using TEM, and the autolysosomes were marked in red arrow. Scale bar = 500 nm. (K) The expression of autophagy‐related proteins was detected by Western blot. (L) LC3B‐GFP‐mCherry plasmids were transfected into OS cells that overexpress ITGBL1 or vector for confocal microscopic analysis to examine the expression of GFP and mCherry. Scale bar = 10 µm. All data are presented as the means ± SD, and differences between groups were assessed by Student's *t*‐test. * *p* < 0.05; ** *p* < 0.01; *** *p* < 0.001; **** *p* < 0.0001.

To investigate the molecular mechanism by which ITGBL1 inhibits OS progression, RNA‐sequencing was performed on 143B cells overexpressing ITGBL1 and control 143B cells (Figure 4A). Kyoto Encyclopedia of Genes and Genomes (KEGG) enrichment analysis and Gene Ontology (GO) enrichment analysis of RNA‐sequencing data revealed that ITGBL1 expression was associated with the endoplasmic reticulum (ER) pathway in OS cells (Figure 4B,C). Gene set enrichment analysis (GSEA) further confirmed that the ITGBL1 level was positively correlated with ER stress in OS cells (Figure 4D). Additionally, Transmission electron microscopy (TEM) analysis revealed greater endoplasmic reticulum thickness in ITGBL1 overexpressing OS cells than in control (Figure 4E). Importantly, our data revealed that ITGBL1 positively regulates ER stress‐related proteins expression such as BIP, PERK, IRE1α, and CHOP (Figure 4F–H). Subsequent confocal microscopy analysis of OS cells transfected with the ITGBL1‐GFP and pDsRed‐ER plasmids demonstrated that ITGBL1 was localized within the ER (Figure 4I). Because ER stress can induce autophagy [21, 33, 34] and ER‐autophagy plays an important role in cancer progression [35, 36], we further investigated whether ITGBL1 is involved in the regulation of autophagy in OS cells. The increased autolysosome formation induced by ITGBL1 overexpression was detected in OS cells using TEM (Figure 4J). Moreover, ITGBL1 positively regulated the expression of LC3B in OS cells, but negatively regulated the expression of p62 (Figure 4K). Increased autophagy by ITGBL1 overexpression was further confirmed in OS cells transfected with the LC3B‐GFP‐mCherry plasmid (Figure 4L). Collectively, these results suggest that ITGBL1 overexpression activates ER stress and autophagy in OS cells.

Given that reactive oxygen species (ROS) are implicated in the induction of ER stress [37], we next asked whether ITGBL1 promotes ER stress in OS by elevating intracellular ROS. Cellular ROS levels were assessed in OS cells with or without ITGBL1 manipulation. Our data suggested that ITGBL1 overexpression markedly increased ROS, whereas treatment with N‐acetylcysteine (NAC), a ROS scavenger, abrogated the ITGBL1‐induced ROS elevation (Figure S3A). Importantly, NAC substantially attenuated ITGBL1‐driven ER stress and autophagy (Figure S3B), indicating that ITGBL1 activates ER stress and autophagy, at least in part, through ROS upregulation in OS cells.

### 3.5 ITGBL1 Suppresses OS Progression Through ER Stress‐Induced Autophagy

**FIGURE 5:**
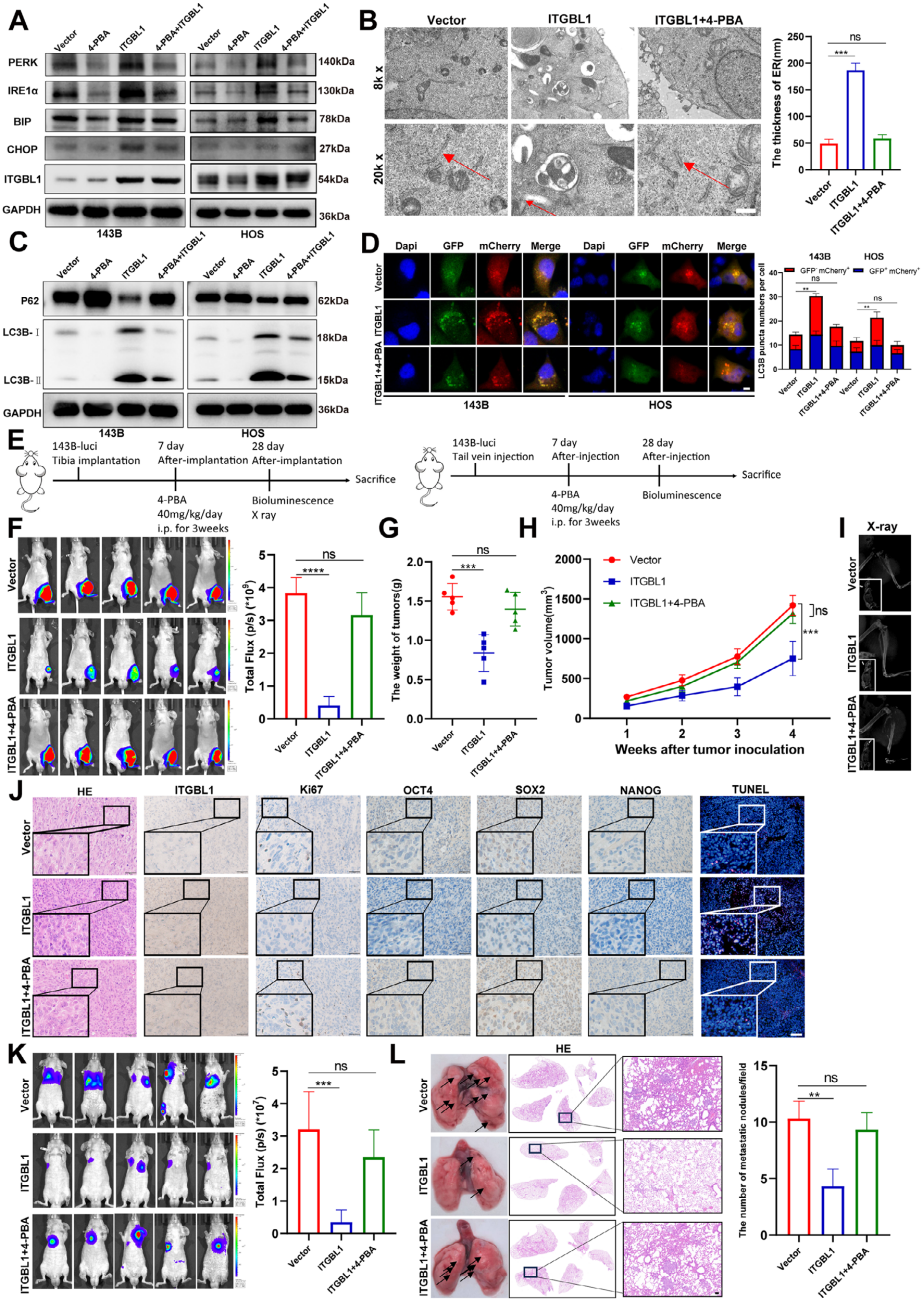
ITGBL1 suppresses OS progression through ER stress. (A) The expression of ER stress‐related proteins was detected by Western blot after overexpressing ITGBL1 or/and administration of ER stress inhibitor 4‐PBA (1 mm) in 143B and HOS. (B) ER was detected by TEM in 143B after overexpressing ITGBL1 with or without 4‐PBA (1 mm for 24 h). Scale bar = 500 nm. (C) The expression of p62 and LC3B was detected by Western blot after overexpressing ITGBL1 or/and administration 4‐PBA in 143B and HOS. (D) LC3B‐GFP‐mCherry plasmids were transfected into OS cells that overexpress ITGBL1 or administered 4‐PBA for confocal microscopic analysis to examine the expression of GFP and mCherry. Scale bar = 10 µm. (E) Schematic diagram for orthotopic OS model and OS lung metastasis model experiments. (F) Bioluminescence images and quantification of orthotopic OS model (n = 5). (G,H) The tumor weight and growth in orthotopic OS models. (I) Typical X‐ray image of tibia in orthotopic OS models. (J) HE, IHC (ITGBL1, Ki67, OCT4, SOX2, and NANOG), and TUNEL staining of orthotopic xenograft tumors. Scale bar = 50 µm in HE and IHC images, Scale bar = 100 µm in TUNEL images. (K) Bioluminescence images and quantification of OS lung metastasis models. (L) Representative images of lung and lung tissue HE staining in OS lung metastasis models. Scale bar = 100 µm. All data are presented as the means ± SD, and differences between groups were assessed by Student's *t*‐test. * *p* < 0.05; ** *p* < 0.01; *** *p* < 0.001; **** *p* < 0.0001.

Next, we investigated whether ITGBL1 plays an anti‐cancer role by activating ER stress‐induced autophagy. In vitro experimental results showed that treatment of the ER stress inhibitor 4‐PBA dramatically suppressed ITGBL1‐induced ER stress activation in OS cells (Figure 5A,B). Importantly, ER stress inhibition significantly attenuated the autophagy‐inducing effect of ITGBL1 in OS cells (Figure 5C,D), as well as the inhibitory effects of ITGBL1 on OS cell proliferation (Figure S4A), metastasis (Figure S4B,C), sphere formation (Figure S4D), and expression of the stemness‐related proteins (Figure S4E). In addition, inhibition of ER stress significantly suppressed ITGBL1 overexpression‐induced apoptosis of OS cells (Figure S4F). These in vitro results were further confirmed in animal models generated using 143B‐luci cells transfected with vector or ITGBL1‐expressing plasmids. As shown in Figure 5E, after 7 days of OS cell inoculation, 4‐PBA (40 mg/kg/day) was administered via intraperitoneal injection for 3 weeks. The results of the orthotopic OS model revealed that 4‐PBA treatment significantly attenuated the inhibitory effects of ITGBL1 on OS growth (Figure 5F–H), bone destruction (Figure 5I), and the expressions of Ki67, OCT4, SOX2, and NANOG, as well as the promoting effect of ITGBL1 on apoptosis (Figure 5J). The inhibition of ER‐stress by 4‐PBA also significantly attenuated the inhibitory effect of ITGBL1 on OS lung metastasis in vivo (Figure 5K,L). Notably, inhibition of autophagy attenuated the ITGBL1‐induced OS growth inhibition in the orthotopic OS model (Figure S5A–E), indicating ITGBL1 suppresses OS progression at least through inducing autophagy. Taken together, our findings indicate that ITGBL1 stimulates autophagy by inducing ER stress, thereby suppressing OS progression.

### 3.6 HSP90AB1 Directly Interacts With ITGBL1 in OS Cells

**FIGURE 6:**
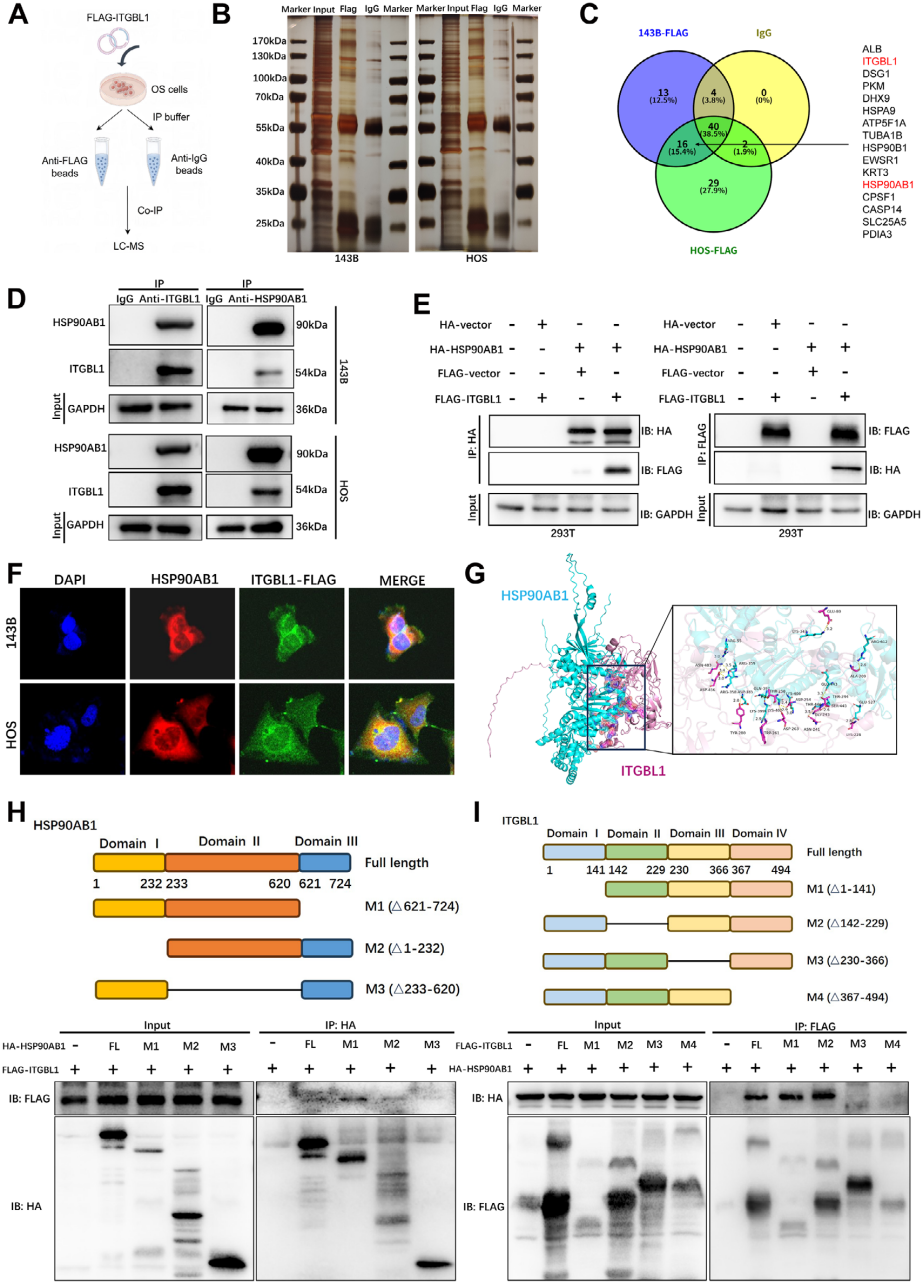
HSP90AB1 directly interacts with ITGBL1 in OS cells. (A) Schematic diagram for Co‐IP/MS analysis in OS cells. (B) Silver staining of proteins that isolated from OS cells by IgG or FLAG antibodies. (C) Fifteen proteins were screened out among the intersection between 143B and HOS anti‐FLAG groups, while ruled out proteins in the anti‐IgG group. (D) Co‐IP analysis was performed with anti‐ITGBL1 or anti‐HSP90AB1 antibodies in 143B and HOS cells. (E) 293T cells were transfected with plasmid that expressing HA‐HSP90AB1 or FLAG‐ITGBL1, and then cell lysates were subjected to Co‐IP assay with anti‐HA or anti‐FLAG antibodies. (F) The subcellular localization of HSP90AB1 and ITGBL1 in 143B and HOS cells was detected by IF staining. (G) The binding sites between HSP90AB1 and ITGBL1 were predicted by Molecular Docking. (H) Schematic representation of HA‐HSP90AB1 truncations was shown (upper panel). 293T cells were co‐transfected with FLAG‐ITGBL1 and the indicated HA‐HSP90AB1 truncations. The cells were lysed for immunoprecipitation using anti‐HA antibody, and anti‐FLAG antibody was used to detect the protein binding (lower panel). (I) A schematic representation of ITGBL1 truncations was shown (upper panel). The truncations of FLAG‐ITGBL1 and full‐length HA‐HSP90AB1 were co‐transfected in 293T cells, followed with Co‐IP analysis.

Our data indicate that ITGBL1 is downregulated in OS, however, the molecular mechanism underlying the downregulation of ITGBL1 in OS is unclear. Here, we performed Co‐IP/MS analysis using 143B and HOS cells transfected with FLAG‐ITGBL1 (Figure 6A). LC‐MS analysis and silver staining (Figure 6B) revealed that numerous proteins interact with ITGBL1 in OS cells. By comparing the protein profiles between the 143B and HOS anti‐FLAG groups while excluding proteins from the anti‐IgG control group, we identified 15 potential candidate proteins that interact with ITGBL1 (Figure 6C). Among them, we chose HSP90AB1 for subsequent experiments, based on the fact that previous studies have shown that HSP90AB1 is involved in the regulation of cancer metastasis [14, 15, 38], apoptosis [39], cancer stemness [40, 41], and ER autophagy [42, 43]. The interaction between ITGBL1 and HSP90AB1 in OS cells was further confirmed by Co‐IP assay using endogenous (Figure 6D) and exogenous proteins (Figure 6E). Immunofluorescence assay also revealed the colocalization of HSP90AB1 and ITGBL1 in OS cells (Figure 6F). To identify the binding domain between HSP90AB1 and ITGBL1, protein– docking analysis was subsequently performed, which revealed that the 209–483 amino acid sequence of ITGBL1 and the 359–612 amino acid sequence of HSP90AB1 may be key sites for their binding to each other (Figure 6G). To determine whether these two proteins bind to each other through these predicted sites, several truncated mutants of HSP90AB1 (Figure 6H) and ITGBL1 (Figure 6I) were generated. 293T cells were co‐transfected with HSP90AB1 mutant and ITGBL1 or ITGBL1 mutant and with HSP90AB1, followed by Co‐IP analysis. The results indicated that domain II of HSP90AB1 (amino acids 233–620) (Figure 6H) and domains III‐IV of ITGBL1 (amino acids 230–494) (Figure 6I) are necessary for the interaction between these two proteins.

### 3.7 HSP90AB1 Promotes ITGBL1 Degradation Through K63‐Linked Ubiquitination

**FIGURE 7:**
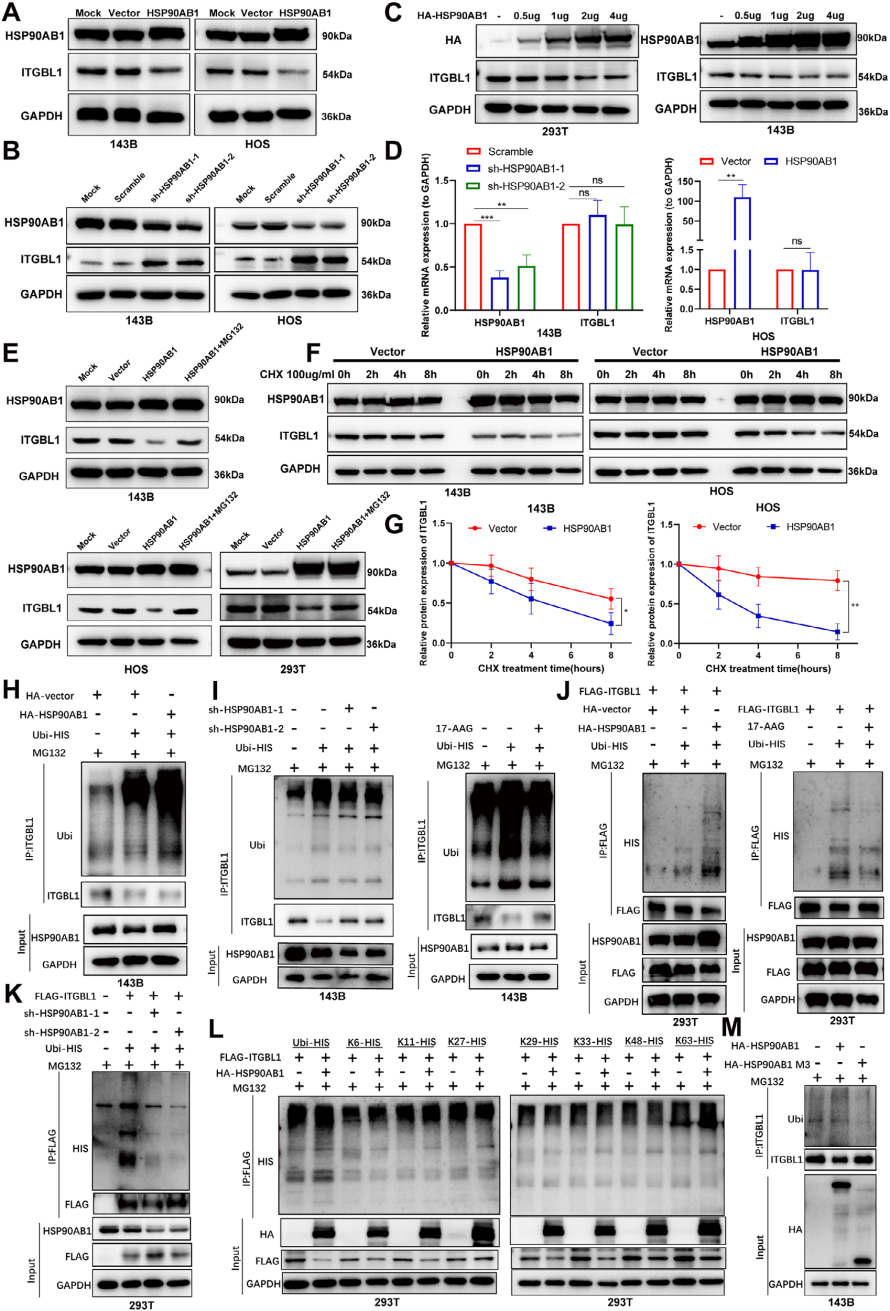
HSP90AB1 stimulates ITGBL1 degradation through K63‐linked ubiquitination. (A,B) The expression of indicated proteins was detected in 143B and HOS cells by Western blot after cells were transfected with the indicated plasmids. (C) The expression of ITGBL1 was detected in OS cells by WB after transfecting different doses of HA‐HSP90AB1. (D) The mRNA expression of ITGBL1 was measured by RT‐qPCR in OS cells after transfection with the indicated plasmids. (E) 143B, HOS, and 293T cells were transfected with HSP90AB1 expressing plasmids, and treated with or without 20 µm MG132 for 9 h, and then assessed ITGBL1 expression. (F,G) OS cells transfected with HSP90AB1 expressing plasmids or vector were treated with 100 µg/mL CHX, and detected the expression levels of indicated proteins by WB at the indicated time points. Then ITGBL1 protein abundance was quantified by the Image J software. (H,I) The ubiquitination of ITGBL1 was analyzed in 143B cells that transfected with the indicated plasmids or treated with 17‐AAG. (J,K) 293T cells were transfected with the indicated constructs or treated with HSP90 inhibitor 17‐AAG. After 48 h of transfection, cells were treated with 20 µm MG132 for 9 h. Lysates were subjected to IP assays, followed by immunoblotting analysis. Anti‐HIS antibody was used to bind HIS‐tagged Ub to indicate ubiquitination. (L) Immunoprecipitation assay was used to analyze the ubiquitination of ITGBL1 in 293T cells transfected with HIS‐Ub (WT, K6, K11, K27, K29, K33, K48, or K63) together with FLAG‐ITGBL1, HA‐HSP90AB1. (M) The ubiquitination of ITGBL1 was analyzed in 143B cells that transfected with the indicated plasmids.

Next, we investigated the effect of HSP90AB1 on ITGBL1 expression. Western blot analysis showed that HSP90AB1 negatively regulates the expression of ITGBL1 in OS cells (Figure 7A,B), and HSP90AB1 induced a dose‐dependent reduction in ITGBL1 protein levels in both 293T and 143B cells (Figure 7C). However, the overexpression or knockdown of HSP90AB1 did not change the mRNA expression levels of ITGBL1 in OS cells (Figure 7D). In addition, that treatment of the proteasome inhibitor MG132 inhibited the degradation of ITGBL1 protein induced by HSP90AB1 (Figure 7E) in OS and 293T cells, whereas the treatment of the protein synthesis inhibitor cycloheximide (CHX) accelerated the degradation of the ITGBL1 protein by HSP90AB1 (Figure 7F,G). These results suggest that HSP90AB1 regulates ITGBL1 expression at the post‐transcriptional level through the proteasomal degradation system. Since the proteasome degrades proteins labeled with ubiquitin, we investigated whether HSP90AB1 is involved in the regulation of ITGBL1 ubiquitination. The results of the ubiquitination assay revealed that the overexpression of HSP90AB1 increased the ubiquitination of ITGBL1 (Figure 7H), whereas the inhibition or knockdown of HSP90AB1 decreased the ubiquitination of ITGBL1 in OS cells (Figure 7I). These results were further confirmed with exogenous proteins (Figure 7J,K). Then, to identify the specific type of ubiquitin linkage involved in HSP90AB1‐regulated ITGBL1 ubiquitination, we transfected various ubiquitin monomers (K6‐HIS, K11‐HIS, K27‐HIS, K29‐HIS, K33‐HIS, K48‐HIS, and K63‐HIS) into 293T cells. Our results demonstrated that HSP90AB1 preferentially recruited K63‐linked ubiquitin chains, which facilitated the degradation of ITGBL1 (Figure 7L). In addition, unlike wild type, the overexpression of the HSP90AB1 mutant, which lacks amino acids 233–620, did not affect the ubiquitination or protein levels of ITGBL1 in OS cells (Figure 7M). Together, these data suggest that HSP90AB1 downregulates ITGBL1 by stimulating K63‐linked ubiquitination‐mediated degradation through direct binding to ITGBL1 in OS cells.

### 3.8 HSP90AB1 Inhibits the Anticancer Effects of ITGBL1 on OS by Decreasing its Expression Level

**FIGURE 8:**
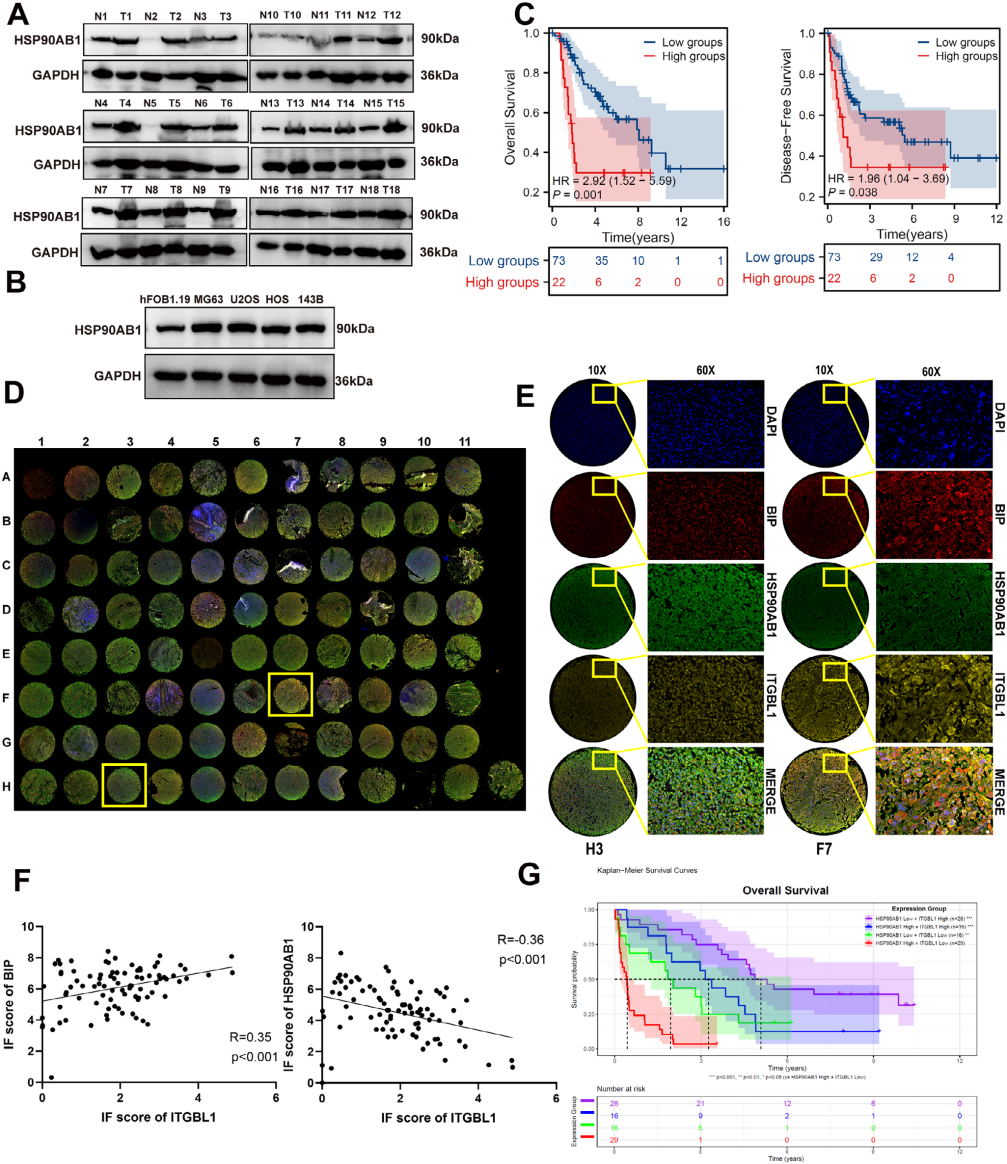
The expression of HSP90AB1 in OS and correlation among ITGBL1 and BIP, HSP90AB1. (A) The protein expression level of HSP90AB1 in OS tissues and their normal bone tissues (*n* = 18 pairs). (B) The protein expression level of HSP90AB1 in the indicated cells. (C) Kaplan–Meier analyses of the overall survival rate and disease‐free survival rate of OS patients with high and low expression levels of HSP90AB1. (D–F) IF staining of BIP, HSP90AB1, and ITGBL1 with OS tissue microarrays and the relationship among ITGBL1 and BIP, HSP90AB1. Scale bar = 20 µm. (G) Kaplan–Meier analyses of the overall survival rate of OS patients with high or low expression levels of ITGBL1 and HSP90AB1.

The above results indicate that HSP90AB1 negatively regulates ITGBL1 expression, suggesting that HSP90AB1 may inhibit the anticancer effect of ITGBL1 in OS. Our in vitro experiments revealed that the overexpression of HSP90AB1 in OS cells suppressed ITGBL1‐induced endoplasmic reticulum dilation (Figure S6A), ER stress (Figure S6B), and autophagy (Figure S6C–E), as well as the inhibitory effects of ITGBL1 on the proliferation (Figure S7A), metastasis (Figure S7B,C), sphere formation (Figure S7D), and expression of the stemness‐related proteins OCT4, SOX2 and NANOG in OS cells (Figure S7E). Additionally, the TUNEL assay revealed that HSP90AB1 overexpression reduced the apoptosis of OS cells induced by ITGBL1 overexpression (Figure S7F). These in vitro results were further confirmed in animal models. The results of orthotopic model experiments revealed that overexpression of HSP90AB1 attenuated the inhibitory effects of ITGBL1 on tumor growth (Figure S8A–C) and bone destruction of tumors (Figure S8D), as well as the expression of stemness‐related protein OCT4, SOX2, and NANOG in OS (Figure S8E). In addition, HSP90AB1 inhibited ITGBL1 overexpression‐induced apoptosis (Figure S8E), and the inhibition of OS cell lung metastasis (Figure S8F,G). Then, we investigated the impact of the interaction between HSP90AB1 and ITGBL1 on the regulation of OS growth mediated by the HSP90AB1‐ITGBL1 axis. The results of the orthotopic OS model indicated that overexpression of mutant HSP90AB1 failed to effectively counteract the tumor‐suppressive effects of ITGBL1, in contrast to wild‐type HSP90AB1 (Figure S9A–C). Likewise, overexpression of mutant ITGBL1 did not abrogate the tumor‐promoting effect of HSP90AB1 (Figure S9A–C), supporting a functional requirement for their interaction in vivo. We next asked whether reduced ITGBL1 expression in human OS is associated with elevated HSP90AB1. Consistent with this possibility, HSP90AB1 expression was significantly increased in OS tissues and cell lines (Figure 8A,B), and higher HSP90AB1 levels were closely associated with poorer patient prognosis (Figure 8C). Importantly, IF staining with OS tissue microarrays demonstrated that the expression of HSP90AB1 was negatively correlated with the expression of ITGBL1 and BIP, while the expression of ITGBL1 was positively correlated with the expression of BIP in human OS (Figure 8D–F). In addition, survival rate analysis showed that among OS patients with high expression of HSP90AB1, patients with low ITGBL1 expression had lower survival rates than those with high ITGBL1 expression (Figure 8G). Collectively, these data indicate that HSP90AB1 binds to ITGBL1 and downregulates its expression, thereby attenuating the tumor‐suppressive effects of ITGBL1 in osteosarcoma.

### 3.9 Ivermectin Suppresses OS Progression by Blocking the Interaction Between HSP90AB1 and ITGBL1

**FIGURE 9:**
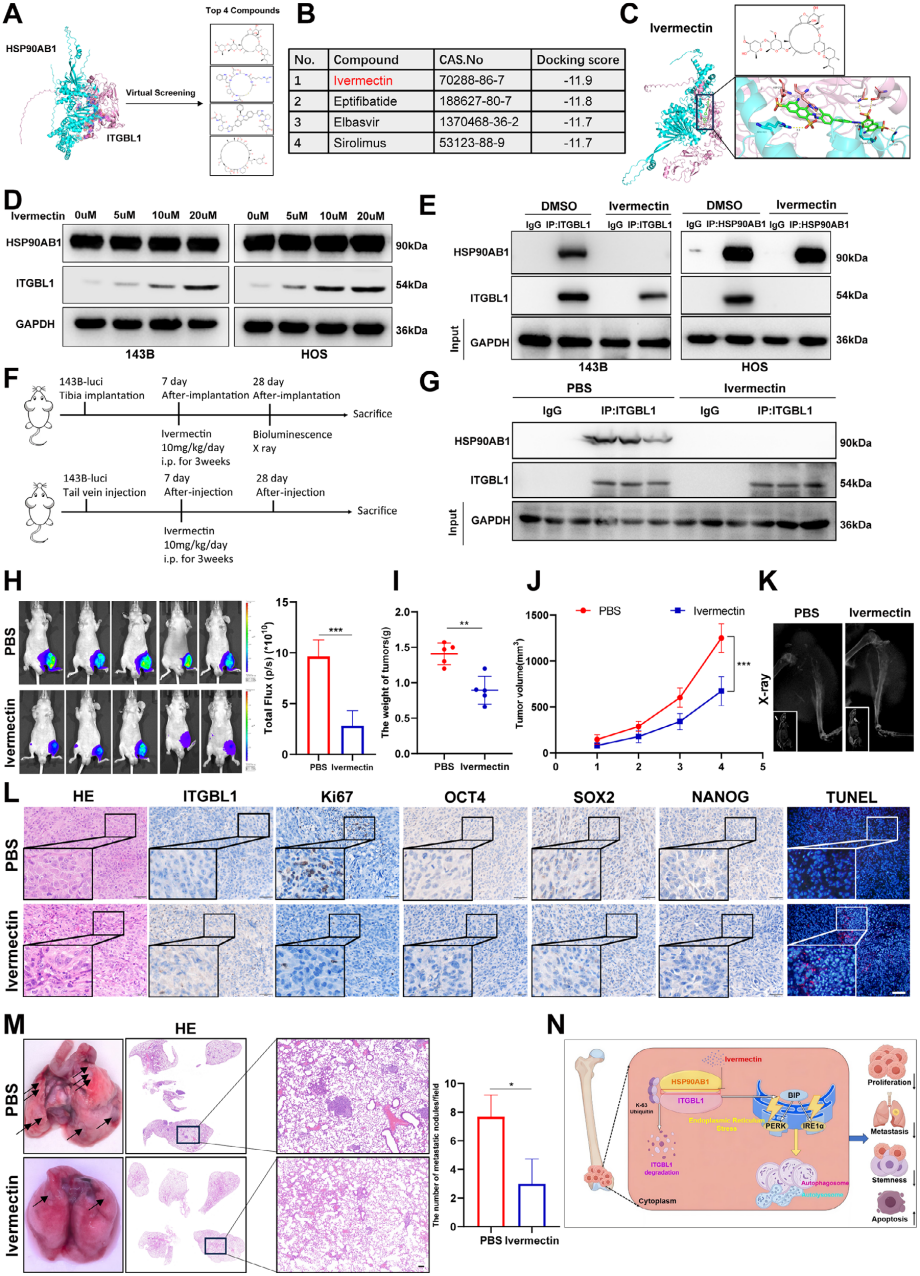
Ivermectin suppresses OS progression by blocking the interaction between HSP90AB1 and ITGBL1. (A,B) Virtual Screening revealed small molecules that targeting the interaction surface between HSP90AB1 and ITGBL1. (C) A diagram showing the structure of ivermectin and the potential binding surface at the HSP90AB1‐ ITGBL1 interaction. (D) Indicated proteins were detected by Western blot in the indicated OS cells that treated with the indicated concentrations of ivermectin for 24 h. (E) Indicated OS cells were treated with DMSO or 20 µm ivermectin for 24 h, then performed Co‐IP using the indicated antibodies. (F) Schematic diagram for an ivermectin treatment animal experiment. (G) Co‐IP analysis was performed using orthotopic xenograft tumors that treated with PBS or Ivermectin. (H) Bioluminescence images and quantification of orthotopic OS model (*n *= 5). (I,J) The tumor weight and growth in orthotopic OS models. (K) Typical X‐ray image of tibia in the orthotopic OS model. (L) HE, IHC (ITGBL1, Ki67, OCT4, SOX2, and NANOG), and TUNEL staining of orthotopic xenograft tumors. Scale bar = 50 µm in HE and IHC images; Scale bar = 100 µm in TUNEL images. (M) Representative images of lung and lung tissue HE staining in OS lung metastasis models. Scale bar = 100 µm. (N) Schematic diagram of HSP90AB1/ITGBL1 axis promoting OS progression (By Figdraw). All data are presented as the means ± SD, and differences between groups were assessed by Student's *t‐*test. * *p* < 0.05; ** *p* < 0.01; *** *p* < 0.001; **** *p* < 0.0001.

The above experimental results indicate that HSP90AB1 interacts with the anticancer protein ITGBL1, leading to the degradation of ITGBL1 and promoting OS progression. These findings suggest that blocking the interaction between HSP90AB1 and ITGBL1 may restore ITGBL1 expression, thereby inhibiting OS progression. Thus, we screened small molecules from 2115 FDA‐approved small molecule compounds in the ZINC database [44] that may block the binding of HSP90AB1 and ITGBL1, and identified four candidate small molecules that targeted the binding sites between HSP90AB1 and ITGBL1 (Figure 9A). Among them, we chose ivermectin for subsequent experiments because it is able to bind at the interface between HSP90AB1 and ITGBL1 and has the lowest binding energy (Figure 9B,C). Our in vitro results revealed that ivermectin treatment did not affect the expression of HSP90AB1, but upregulated ITGBL1 expression (Figure 9D), and inhibited the interaction between HSP90AB1 and ITGBL1 in OS cells (Figure 9E). Importantly, in vitro results revealed that ivermectin treatment significantly inhibited OS cell proliferation (Figure S10A), migration and invasion (Figure S10B), and stemness (Figure S10C), while promoting OS apoptosis (Figure S10D), ER‐stress, and autophagy (Figure S10E). These anti‐OS functions of ivermectin were dramatically blocked by the knockdown of ITGBL1 in vitro (Figure S10A–E), suggesting that ivermectin plays an anti‐OS role through ITGBL1 by blocking the interaction of HSP90AB1 and ITGBL1. The anti‐OS effects of ivermectin were further confirmed in animal models. After establishing an orthotopic OS model and an OS lung metastasis model with 143B‐luci cells, ivermectin was given at 10 mg/kg/day through intraperitoneal injections for 3 weeks after one week of OS cell implantation (Figure 9F). Co‐IP analysis from cell lysates harvested from xenograft tumors showed that ivermectin significantly inhibited the interaction of HSP90AB1 and ITGBL1 in vivo (Figure 9G). Notably, orthotopic OS model experiment results showed that ivermectin treatment significantly inhibited tumor growth (Figure 9H–J), bone destruction (Figure 9K), and the expression of Ki67, OCT4, SOX2, and NANOG, and significantly promoted the expression of ITGBL1 and apoptosis of OS cells (Figure 9L). The significant inhibitory effects of ivermectin on OS lung metastasis were also observed in the OS lung metastasis model (Figure 9M). Together, these results suggest that ivermectin is a small molecule that blocks the interaction of HSP90AB1 and ITGBL1, and exhibits a strong inhibitory effect on the growth and metastasis of OS.

## 4 Discussion

A major contributor to the high mortality rate of patients with advanced OS is the lack of targeted drugs for OS, and one of the main obstacles to the development of new drugs for OS is the unclear molecular mechanisms that promote OS progression. Here, through a series of in vitro, in vivo experiments and clinical sample analysis, we demonstrated that aberrant overexpression of HSP90AB1 promotes OS progression by inhibiting the ITGBL1/ER stress/autophagy axis. In addition, we revealed that the small molecule ivermectin can block the inhibitory effect of HSP90AB1 on ITGBL1/ER stress/autophagy signaling, thereby dramatically suppressing the progression of OS. Together, we identified a novel molecular mechanism that promotes OS progression and a candidate small molecule drug that can inhibit OS progression.

The abnormal expression of ITGBL1 has been demonstrated in various cancers [6, 45, 46], however, the expression and function of ITGBL1 are opposite in different cancers [5, 10]. Also, ITGBL1 functions in a manner distinct from integrins, which are cell‐surface receptors that bind extracellular matrix components to initiate intracellular signaling and regulate a broad range of cellular processes [5, 47]. Through analysis of our clinical samples and datasets from the TARGET and GEO databases, we demonstrated that ITGBL1 is downregulated in OS, especially in OS patients with lung metastasis, and that low expression of ITGBL1 is closely related to the poor prognosis of OS patients. In addition, our in vitro and animal experiments revealed that the knockdown of ITGBL1 stimulates OS growth, metastasis, and cancer stemness and inhibits apoptosis. In contrast, the overexpression of ITGBL1 dramatically inhibits OS growth and metastasis. Collectively, we demonstrated for the first time that ITGBL1 is abnormally downregulated in OS, and its downregulation is associated with poor prognosis in OS. Our data also indicated that ITGBL1 plays an anticancer role in OS.

Next, we elucidated the anti‐OS molecular mechanism of ITGBL1. Through bioinformatic analysis of the mRNA sequencing results, we found that ITGBL1 is involved in the regulation of ER signaling in OS. In subsequent in vitro experiments, we used WB, IF, and TEM analyses to demonstrate that ITGBL1 induces ER stress by upregulating ROS, thus stimulating autophagy in OS cells. Interestingly, in human cancers, ER stress‐driven autophagy can function as either pro‐survival or pro‐death in a context‐dependent manner [48, 49]. Here, our results show that inhibition of ER‐stress or autophagy significantly suppresses the anti‐OS effect of ITGBL1, suggesting that ITGBL1‐induced ER stress‐driven autophagy functions as pro‐death in OS. Our findings indicate that ITGBL1 plays an anti‐OS role by activating ROS/ER stress/autophagy signaling. To our knowledge, our study reports for the first time that ITGBL1 acts as a positive regulator of the ROS/ER stress/autophagy pathway in OS.

In addition, we elucidated the molecular mechanism underlying the low expression of ITGBL1 in OS. Among the 18 OS patients we analyzed, all patients had lower protein levels of ITGBL1 in their OS tissues compared to normal bone tissues (Figure 1B), but only 12 patients had lower mRNA levels of ITGBL1 in their OS tissues compared to normal bone tissues (Figure 1A). In addition, the mRNA level of ITGBL1 in U2OS cells was higher than that in other OS cell lines (Figure 1C), but the protein expression level of ITGBL1 in U2OS cells is lower than that in other OS cell lines (Figure 1D), suggesting that the expression of ITGBL1 in OS is regulated by both transcriptional and post‐transcriptional mechanisms. Here, we using Co‐IP, MS, IF, and ubiquitination analyses revealed that HSP90AB1 interacts with ITGBL1 and promotes the degradation of the ITGBL1 protein through the K63‐linked ubiquitination pathway, thus causing low expression of ITGBL1 in OS. Notably, the expression of HSP90AB1 is abnormally upregulated in human OS, and the expression of HSP90AB1 and ITGBL1 is negatively correlated. These data indicate that the abnormally upregulated HSP90AB1 promotes the degradation of ITGBL1 protein through the ubiquitination pathway, which is an important post‐translational regulatory mechanism leading to the downregulation of ITGBL1 in OS. However, our study has two limitations. The first limitation is that although the analysis of clinical samples shows that down‐regulation of ITGBL1 in OS caused by the transcriptional and post‐translational mechanisms. But, a limited number of clinical samples (*n* = 18) cannot effectively prove the importance of post‐translation regulation on the abnormal down‐regulation of ITGBL1 in OS, which needs further verification in a large number of clinical samples. The second limitation of this study is that we did not elucidate the transcriptional regulatory molecular mechanism of ITGBL1 in OS. In fact, there are OS patients with low expression of ITGBL1 mRNA, and the low expression of ITGBL1 mRNA is closely correlated to poor prognosis of OS, indicates that future research needs to elucidate the molecular mechanism underlying the low expression of ITGBL1 mRNA in OS.

Additionally, the inhibition of ITGBL1 expression by HSP90AB1 through the ubiquitination pathway is also a novel molecular mechanism by which HSP90AB1 plays an oncogenic role. Previous studies have shown that HSP90AB1 stimulates metastasis in various cancers by upregulating stemness [14], and inhibiting ER stress [42] and autophagy [50]. Notably, HSP90AB1 is an important therapeutic target in cancer [51]. In addition, an important feature of HSP90AB1 is that HSP90AB1 plays its cancer‐promoting roles through interaction with other proteins [14, 42]. Here, we demonstrated that HSP90AB1 stimulates OS cells proliferation, metastasis, stemness, and inhibits apoptosis, ER stress and autophagy by downregulating ITGBL1 through direct binding to ITGBL1. Importantly, overexpression of ITGBL1 or blocking the interaction between HSP90AB1 and ITGBL1 dramatically inhibits HSP90AB1 overexpression‐induced OS progression. Collectively, we have demonstrated a novel molecular mechanism by which HSP90AB1 promotes OS progression and proposed a novel therapeutic strategy for treating OS with high expression of HSP90AB1.

Finally, our mechanistic insights prompted an exploration of translational applications. The molecular chaperone HSP90 has long been pursued as a cancer therapeutic target, yet the clinical advancement of pan‐HSP90 ATP‐competitive inhibitors (e.g., pimitespib, the sole approved agent in this class [52]) has been constrained by substantial limitations. These include dose‐limiting toxicities arising from the simultaneous destabilization of a vast array of HSP90 client proteins, the induction of a compensatory heat shock response, and a lack of tumor specificity [53]. These challenges are inherent to the pharmacologic strategy of global chaperone inhibition. To circumvent these limitations, here, we identified a small molecule that inhibits the binding of ITGBL1 and HSP90AB1 and has strong anti‐OS effects. Through virtual screening and experimental validation, we demonstrated that ivermectin, approved by the FDA as an antiparasitic agent [54], can block the interaction between ITGBL1 and HSP90AB1 by targeting their binding sites, and restore the expression of ITGBL1 in OS. Importantly, ivermectin significantly inhibited OS growth and lung metastasis in animal models. Interestingly, recent studies have shown that ivermectin plays strong anticancer effects on various cancers through inhibiting several cancer‐promoting pathways including the Wnt pathway, the Akt pathway, the MAPK pathway, and the YAP pathways [55, 56, 57, 58, 59]. Importantly, these oncogenic signaling pathways inhibited by ivermectin are also abnormally activated in OS [60, 61, 62]. These findings indicate that ivermectin is a promising OS‐targeted drug.

In summary, abnormally upregulated HSP90AB1 downregulates ITGBL1 expression by promoting ITGBL1 degradation through the activation of K63‐linked ubiquitination, and the downregulation of ITGBL1 leads to the inactivation of ER stress and autophagy, thus promoting tumor growth, metastasis, stemness and inhibiting apoptosis, ultimately leading to the progression of OS (Figure 9N). Ivermectin treatment can inhibit OS progression by activating ER‐autophagy by disrupting the interaction between HSP90AB1 and ITGBL1.

## Author Contributions

M.X., X.C.X., and S.C.W. devised the concept and designed the study. Z.W. and W.C.C. carried out the most experiments. Z.W. and H.Z. finished the bioinformatic analysis. Z.W., X.C.X., S.C.W., X.Z.G., T.X.J., and Y.Z.W. contributed to the manuscript writing. F.Z.H., J.X.Z., S.Y.Z., K.D.S., F.L.Y., L.X.Z., Q.D.X., S.L., and X.H. provided advice. All authors reviewed the manuscript and signed off on its accuracy.

## Funding

This work was supported by the National Key Research and Development Program of China (2023YFB4706305, to M.X.), National Defense Science and Technology Excellence Youth Science Fund Program (2022JCJQ‐ZQ‐018, to M.X.), the Chongqing Natural Science Foundation (CSTB2022NSCQ‐MSX0362, to X.C.X.), and the National Natural Science Foundation of China (82573393, S.C.W.).

## Disclosure Statement

We declare that we have no financial and personal relationships with other organizations that can inappropriately influence our work, and there is no professional or other personal interest of any nature or kind in any product, service, or company that could be constructed as influencing the position presented in, or the review of, the manuscript entitled.

## Ethics Statement

The studies involving human participants were reviewed and approved by the Ethics Committee of Chinese PLA General Hospital. The patients/participants provided their written informed consent to participate in this study. And the care of the animals involved in the experiments was in accordance with the institution's guidelines.

## Conflicts of Interest

The authors declare no conflicts of interest.

## Supporting information




**Supporting File 1**: advs74384‐sup‐0001‐SuppMat.docx.

**Supporting File 2**: advs74384‐sup‐0002‐SuppRawData.pptx.

## Data Availability

The data generated in this study are available upon request from the corresponding author.
