# Supplementary material for: HSP90AB1‐Mediated Ubiquitin‐Proteasome Degradation of ITGBL1 Promotes Osteosarcoma Progression by Inhibiting Endoplasmic Reticulum Stress‐Induced Autophagy
Source: Adv Sci (Weinh). 2026 Feb 16;13(23):e15651. doi: 10.1002/advs.202515651 (PMC13104077; doi:10.1002/advs.202515651)
Supplement: Supplementary file 1 — Supporting File 1: advs74384‐sup‐0001‐SuppMat.docx. [file ADVS-13-e15651-s001.docx]

**HSP90AB1-Mediated Ubiquitin-Proteasome Degradation of ITGBL1 Promotes Osteosarcoma Progression by Inhibiting Endoplasmic Reticulum Stress Induced Autophagy**

**Zhen Wang^1,2^, Zixuan Guo^1,2^, Chengwei Cao^1,2^, Ziying Wang^1,2^, Zifu Huang ^1,2^, Xiujuan, Zhang^3^, Yushu Zheng^1,2^, Diankun She^4^, Hao Zhu^5^, Lingfeng Yu^6^, Xuelin Zhao^1,2^, Dongquan Xiang^2^, Song Liao^2^, Xin He^7^, Xintong Ji ^8^, Chengsheng Wu^7*^, Cheng-Xiong Xu^8*^, Meng Xu^1,2*^**

^1^Medical School of Chinese PLA, Beijing, China

^2^Senior Department of Orthopedics, the Fourth Medical Centre, Chinese PLA General Hospital, Beijing, China

^3^Department of Cell Engineering, Beijing Institute of Biotechnology, Beijing, China

^4^Department of Orthopedics, Jinling Hospital, Affiliated Hospital of Medical School, Nanjing University, Nanjing, Jiangsu, China

^5^Institute of Basic Medical Sciences Chinese Academy of Medical Sciences, School of Basic Medicine Peking Union Medical College, Beijing, China

^6^Department of Orthopedic Oncology, Shanghai Bone Tumor Institute, Shanghai General Hospital, Shanghai Jiao Tong University School of Medicine, Shanghai, China

^7^College of Life Sciences, University of Chinese Academy of Sciences, Beijing, China

^8^School of Medicine, Chongqing University, Chongqing, China

**^*^Correspondence**

Meng Xu, Fucheng Road 51, Haidian District, Beijing 100853, China.

Email: profxum301@163.com

Cheng-Xiong Xu, Yubei Road 131, Shapingba District, Chongqing 400030, China

Email: xuchengxiong@cqu.edu.cn

Chengsheng Wu, Yanqi Road 1#, Huairou District, Beijing, 100049, China

Email: wuchengsheng@ucas.ac.cn

**Table S1:** **The sequences of shRNAs**

| **sh-ITGBL1-1** | **GCAAGAATTCACAAGACATCATTCAAGAGATGATGTCTTGTGAATTCTTGC** |
| --- | --- |
| **sh-ITGBL1-2** | **GCTGGTTGGCATGGAGATAAATTCAAGAGATTTATCTCCATGCCAACCAGC** |
| **sh-ITGBL1-3** | **GCTGTCTATGACCGATATTCTTTCAAGAGAAGAATATCGGTCATAGACAGC** |
| **sh-HSP90AB1-1** | **CGCATGGAAGAAGTCGATTAGTTCAAGAGACTAATCGACTTCTTCCATGCG** |
| **sh-HSP90AB1-2** | **GGAACGAGAGAAGGAAATTAGTTCAAGAGACTAATTTCCTTCTCTCGTTCC** |
| **sh-HSP90AB1-3** | **GGAAGAGAAAGGTGAGAAAGATTCAAGAGATCTTTCTCACCTTTCTCTTCC** |

**Table S2:** **The sequences of the primers used in the present study**

| **ITGBL1 Forward primer** | **GAGAGCATCAGGAAGTGCCA** |
| --- | --- |
| **ITGBL1 Reverse primer** | **CTCCACAGACCACACCATCG** |
| **HSP90AB1 Forward primer** | **CAGCTTTTGTGGAGCGAGTG** |
| **HSP90AB1 Reverse primer** | **AGTACTCGTCAATGGGCTCG** |
| **BIP Forward primer** | **CACTCCTGAAGGGGAACGTC** |
| **BIP Reverse primer** | **TCAAAGACCGTGTTCTCGGG** |
| **GAPDH Forward primer** | **GGAGCGAGATCCCTCCAAAAT** |
| **GAPDH Reverse primer** | **GGCTGTTGTCATACTTCTCATGG** |

**Table S3: The antibodies we used in the experiments**

| **Anti-ITGBL1 Ab** | **Abcepta, AP8781c** |
| --- | --- |
| **Anti-ITGBL1 Ab** | **Proteintech, 30010-1-AP** |
| **Anti-OCT4 Ab** | **Proteintech, 11263-1-AP** |
| **Anti-SOX2 Ab** | **Proteintech, 11064-1-AP** |
| **Anti-NANOG Ab** | **Proteintech, 14295-1-AP** |
| **Anti-Ki67 Ab** | **Servicebio, GB111499-100** |
| **Anti-HSP90AB1 Ab** | **Proteintech, 11405-1-AP** |
| **Anti-HSP90** **beta Ab** | **Abcam, ab203085** |
| **Anti-FLAG Ab** | **Proteintech, 66008-4-Ig and 20543-1-AP** |
| **Anti-HA Ab** | **Proteintech, 51064-2-AP** |
| **Anti-HIS Ab** | **Proteintech, 66005-1-Ig** |
| **Anti-UBI Ab** | **CST, 3936** |
| **ER Stress Antibody Sampler Kit** | **CST, 9956** |
| **Anti-P62 Ab** | **Proteintech, 18420-1-AP** |
| **Anti-LC3 Ab** | **Proteintech, 14600-1-AP** |
| **Alexa Fluor® 488** | **Abcam,** **ab150113** |
| **Alexa Fluor® 594** | **Abcam,** **ab150080** |
| **GAPDH Rabbit mAb** | **Proteintech, 10494-1-AP** |
| **Goat Anti-Rabbit IgG secondary Ab** | **Proteintech,** **SA00001-2** |
| **Goat Anti-Mouse IgG secondary Ab** | **Proteintech,** **SA00001-1** |


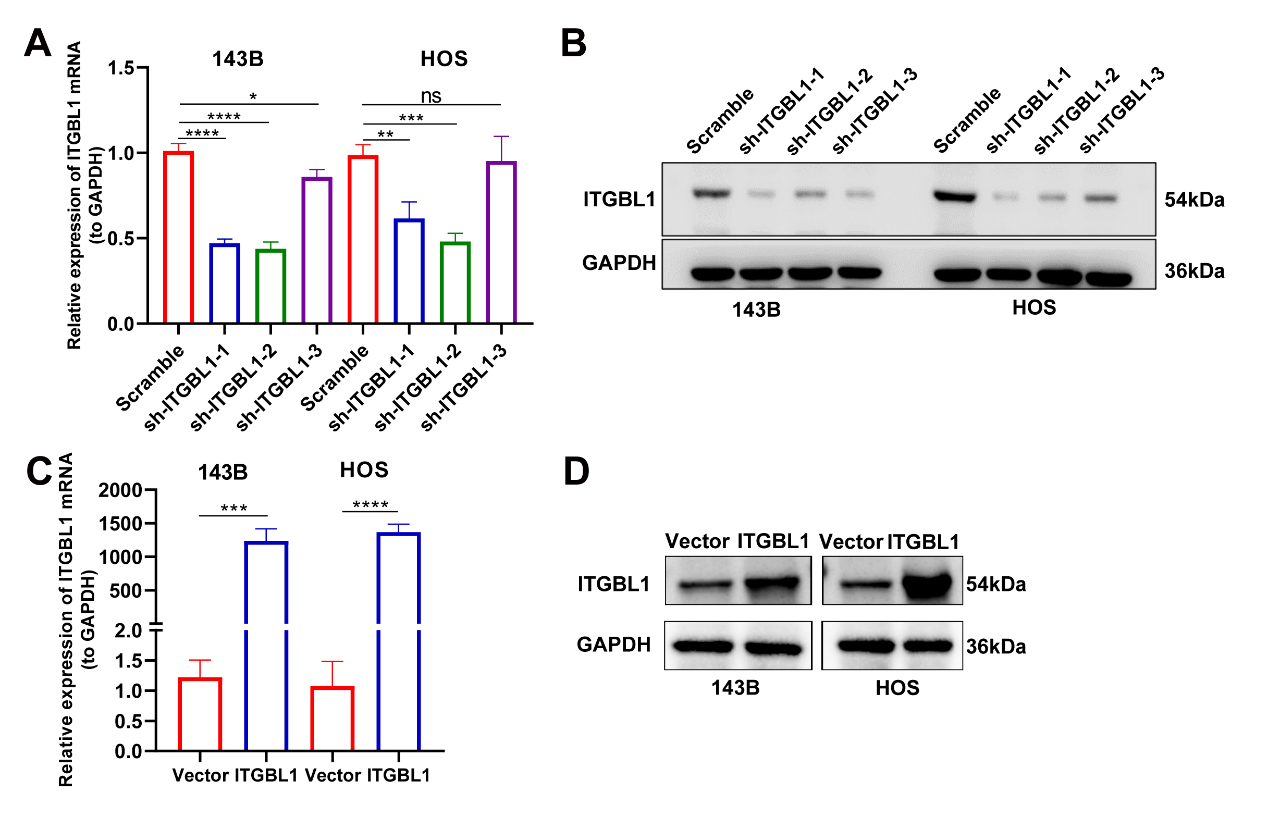


**Figure S1. The efficiency of plasmids expressing ITGBL1 or shRNA of ITGBL1. A-B**. The expression of ITGBL1 was measured by RT-qPCR and Western blot in 143B and HOS cells after transfecting sh-ITGBL1 plasmids. **C-D**. The expression of ITGBL1 was measured by RT-qPCR and Western blot in OS cells after transfecting ITGBL1 expression plasmids. All data are presented as the means ± SD, and differences between groups were assessed by student’s t test. *, *p* < 0.05; **, *p* < 0.01; ***, *p* < 0.001; ****, *p* < 0.0001.


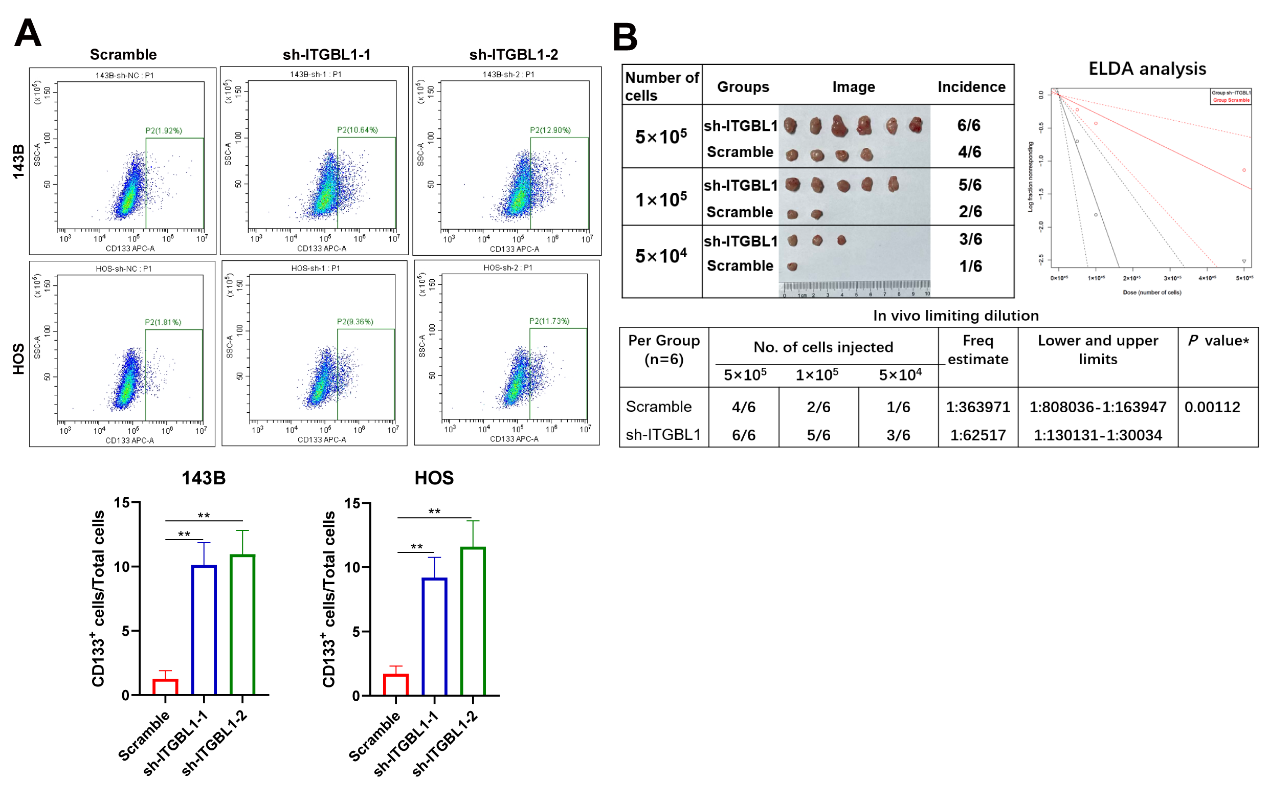


**Figure S2. Downregulation of ITGBL1 promotes OS stemness. A**. CD133 positive cells were detected in OS cells by Flow cytometry after knocking down ITGBL1. **B**. Representative Tumor images from the limiting dilution assay. Indicated number of OS cells transfected with ITGBL1 shRNA or scramble were subcutaneously injected into the back of the nude mice (n=6 per group). CSC frequency analysis was performed using ELDA software. All data are presented as the means ± SD, and differences between groups were assessed by student’s t test. *, *p* < 0.05; **, *p* < 0.01; ***, *p* < 0.001; ****, *p* < 0.0001.


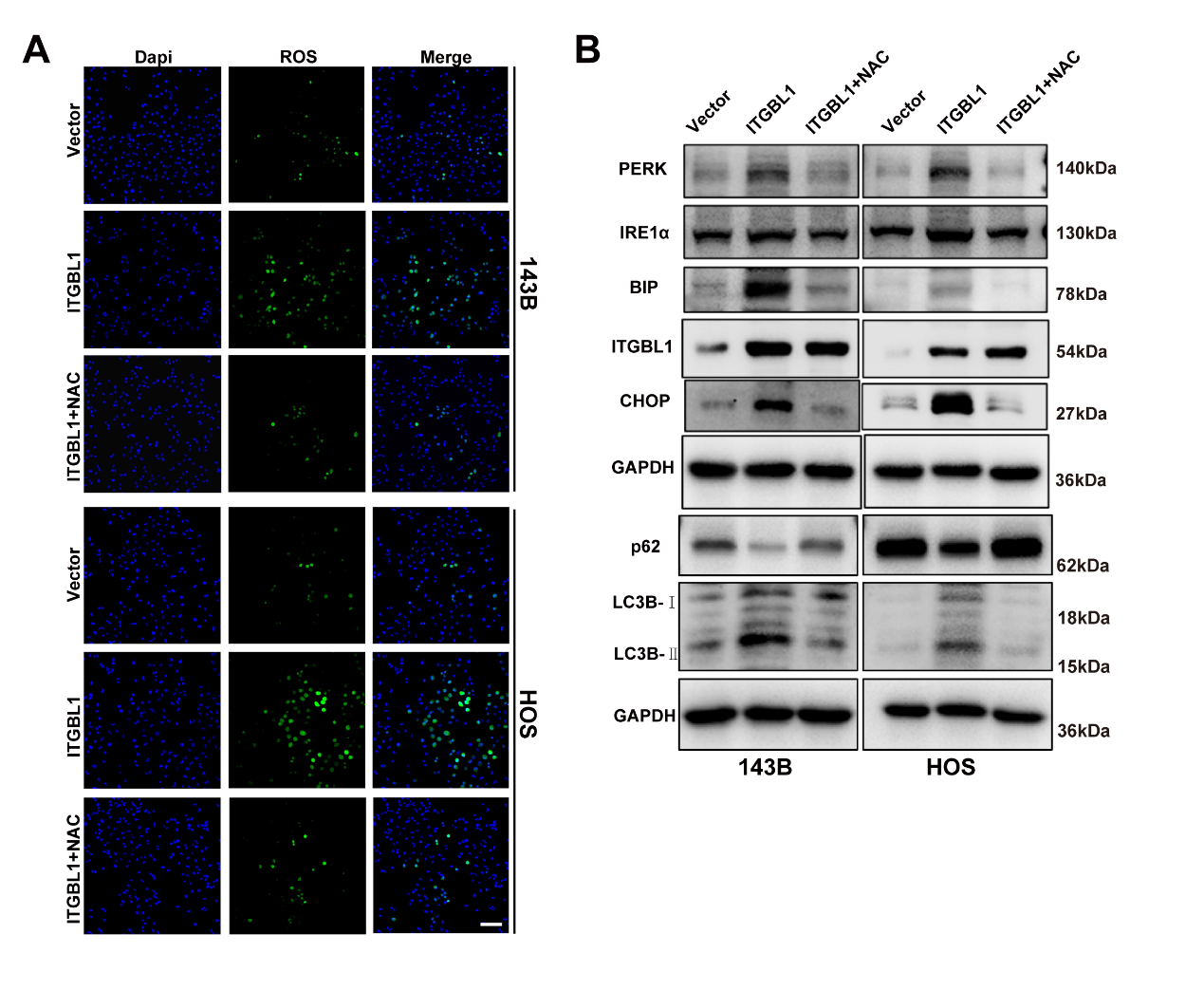


**Figure S3. ITGBL1 overexpression induces ER Stress and autophagy in OS cells by increasing ROS. A**. Intracellular ROS level was detected by DCFH-DA probe after overexpressing ITGBL1 or/and administration of ROS inhibitor N-Acetylcysteine (NAC 5mM) in 143B and HOS. Scale bar = 100 μm. **B**. The expression of ER stress and autophagy related proteins were detected in 143B and HOS by Western blot after overexpressing ITGBL1 or/and administration NAC.


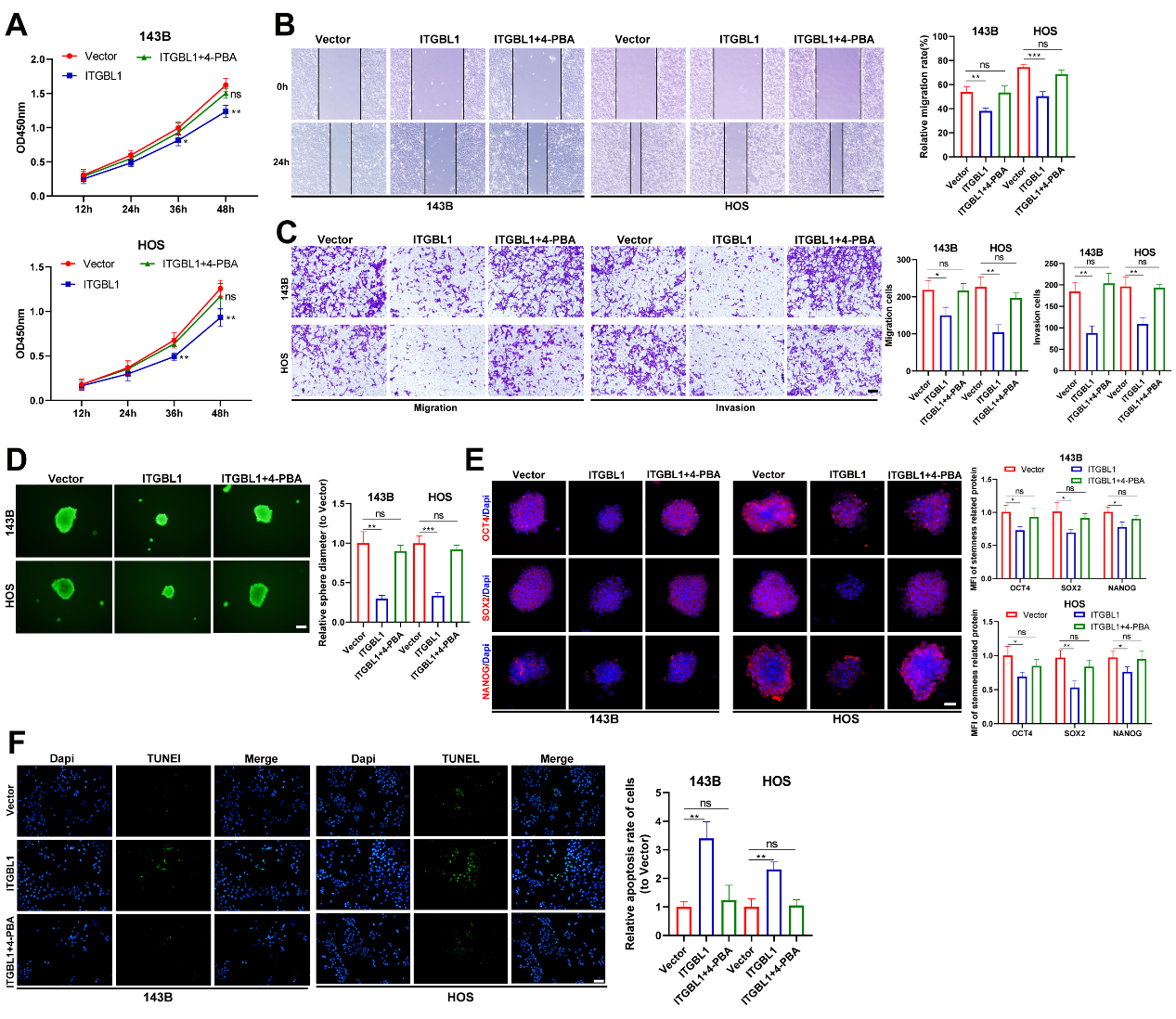


**Figure S4. ITGBL1 suppresses OS progression through ER stress *in vitro*. A**. CCK8, **B**. Wound healing, **C**. Transwell (Scale bar = 100 μm), and **D**. Sphere formation assays were performed to investigate the effects of ER stress inhibition on the anti-OS effects of ITGBL1 (scale bar = 50 μm). **E**. The expression of stemness related protein was detected by immunofluorescence. Scale bar = 50 μm. **F**. TUNEL assay was performed to investigate the effects of ER stress inhibition on the ITGBL1 induced apoptosis. Scale bar = 100 μm. All data are presented as the means ± SD, and differences between groups were assessed by student’s t test. *, *p* < 0.05; **, *p* < 0.01; ***, *p* < 0.001; ****, *p* < 0.0001.


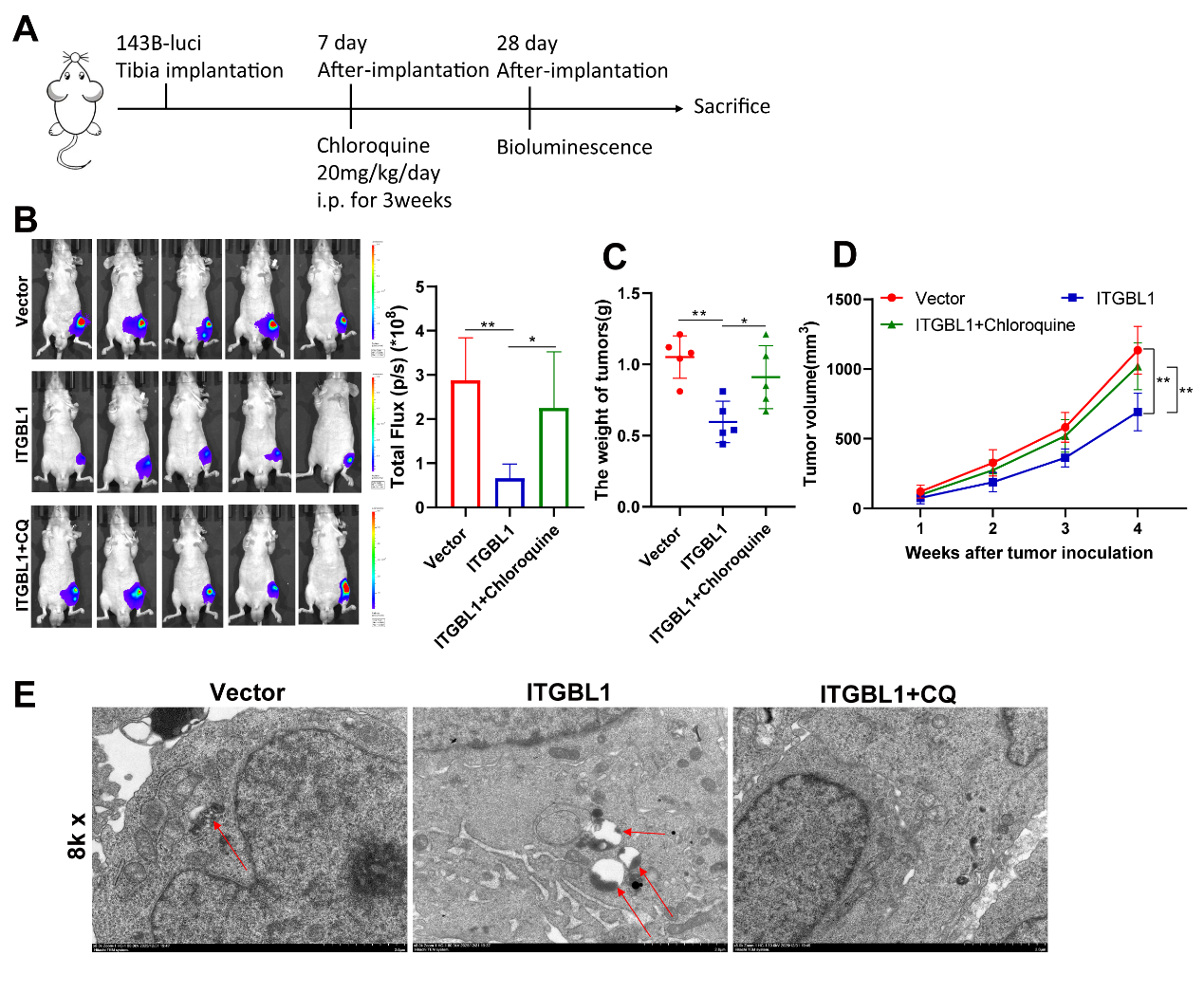


**Figure S5. Administration of autophagy inhibitor Chloroquine (CQ) inhibits the anti-tumor effect of ITGBL1 *in vivo*. A**. Schematic diagram for Chloroquine treatment animal experiment. **B**. Bioluminescence images and quantification of orthotopic OS model (n = 5). **C-D**. The tumor weight and growth in orthotopic OS models. **E.** The autolysosomes in orthotopic xenograft tumors were detected using TEM. All data are presented as the means ± SD, and differences between groups were assessed by student’s t test. *, *p* < 0.05; **, *p* < 0.01; ***, *p* < 0.001; ****, *p* < 0.0001.


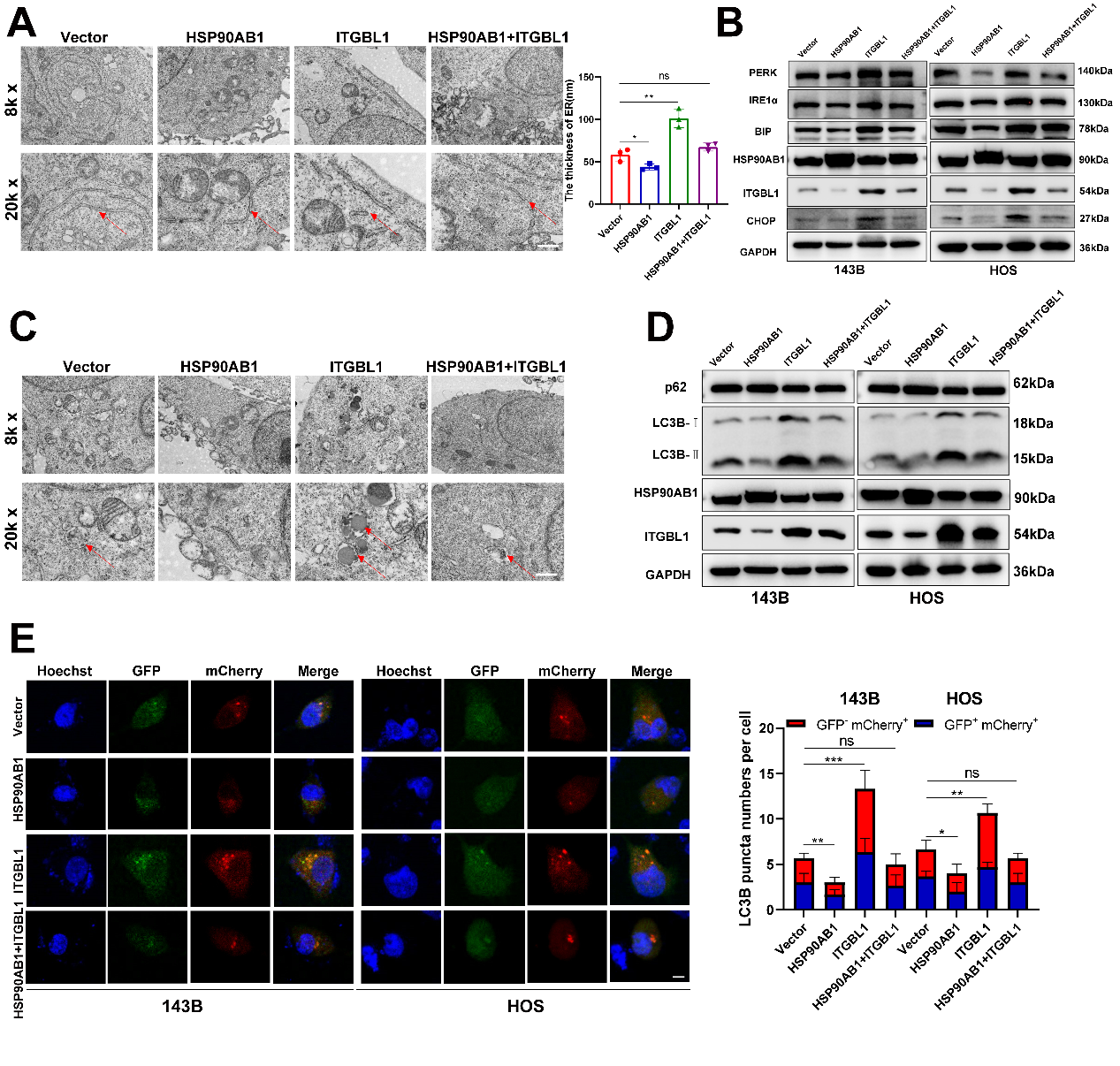


**Figure S6. HSP90AB1 inhibits ITGBL1 induced ER Stress and autophagy in OS cells. A.** The endoplasmic reticulum (ER) was observed by transmission electron microscopy (TEM) in 143B cells after transfecting with HSP90AB1 or/and ITGBL1 expression plasmids. The ER was indicated by red arrow. Scale bar = 500nm. **B**. The expression of ER stress related proteins was detected by Western blot after transfecting with HSP90AB1 or/and ITGBL1 expression plasmids in indicated cells. **C**. Autolysosomes were observed by TEM in 143B cells after transfecting with HSP90AB1 or/and ITGBL1 expression plasmids. Autolysosomes were marked in red arrow. Scale bar = 200nm. **D**. The expression of autophagy related proteins was detected by Western blot in indicated OS cells after transfecting with HSP90AB1 or/and ITGBL1 expression plasmids. **E**. Transfection with HSP90AB1 or/and ITGBL1 expression plasmids into OS cells expressing LC3B-GFP-mCherry, the expression of GFP and mCherry was observed using confocal microscopy. Scale bar = 10μm. All data are presented as the means ± SD, and differences between groups were assessed by student’s t test. *, *p* < 0.05; **, *p* < 0.01; ***, *p* < 0.001; ****, *p* < 0.0001.


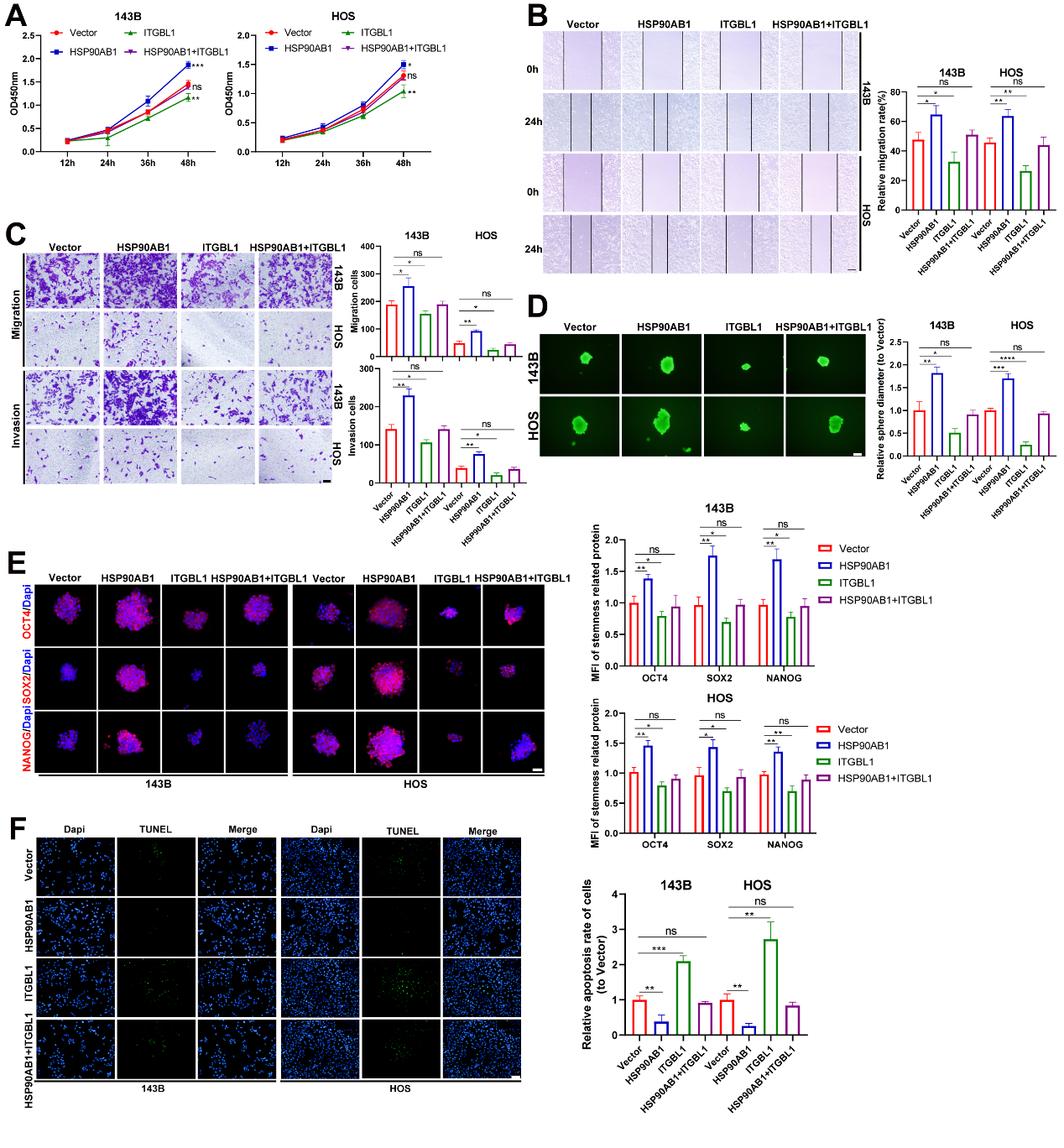


**Figure S7. HSP90AB1 attenuates the inhibitory effects of ITGBL1 on the progression of OS *in vitro***. **A**. CCK8, **B**. Wound healing, **C**. Transwell, and **D**. Sphere formation assays were performed using OS cells that transfected with indicated plasmids. **E**. The expression of stemness-related proteins in OS spheres was measured by immunofluorescence. **F**. TUNEL assays were performed using indicated cells that transfected with indicated plasmids. Scale bar = 100 μm for wound healing, transwell, and TUNEL images; Scale bar = 50 μm for sphere images. All data are presented as the means ± SD, and differences between groups were assessed by student’s t test. *, *p* < 0.05; **, *p* < 0.01; ***, *p* < 0.001; ****, *p* < 0.0001.


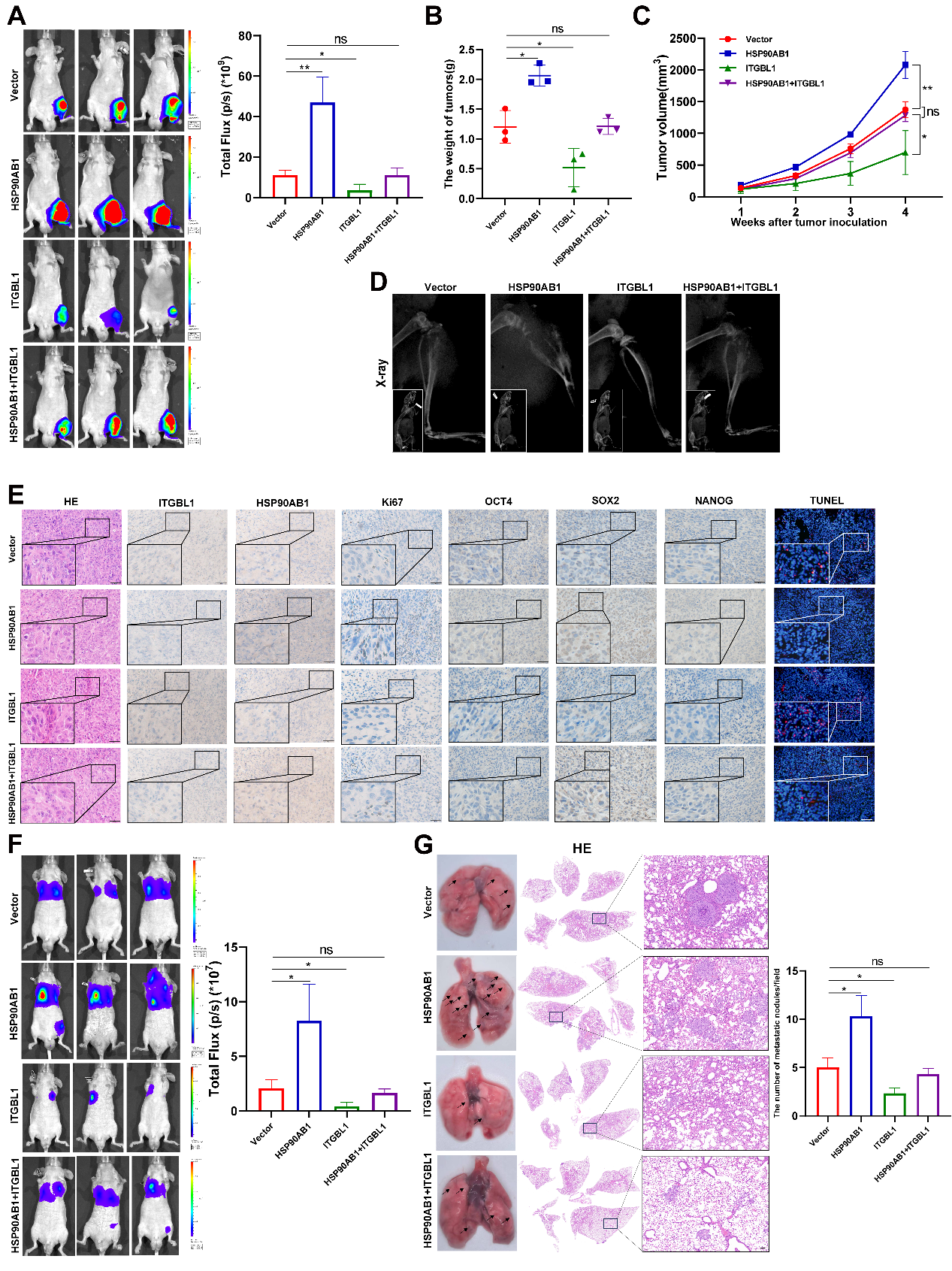


**Figure S8. HSP90AB1 attenuates the inhibitory effects of ITGBL1 on OS progression *in vivo***. **A**. Bioluminescence images and quantification in orthotopic OS models (n = 3 per group). **B-C**. Tumor weight and growth in orthotopic OS models. **D**. X-ray image of tibia in orthotopic OS models. **E**. HE, IHC (ITGBL1, HSP90AB1, Ki67, OCT4, SOX2 and NANOG) and TUNEL staining of orthotopic xenograft tumors. IHC staining was imaged at 200× magnification and scale bar = 50 μm; TUNEL staining was imaged at 100× magnification, and scale bar = 100 μm. **F**. Bioluminescence images and quantification in OS lung metastasis models. **G**. The lung specimen and HE staining of metastatic nodules on lung surface. Scale bar = 100 μm. Orthotopic or OS lung metastasis models were constructed using 143B-luci OS cells that transfected with indicated plasmids. All data are presented as the means ± SD, and differences between groups were assessed by student’s t test. *, *p* < 0.05; **, *p* < 0.01; ***, *p* < 0.001; ****, *p* < 0.0001.


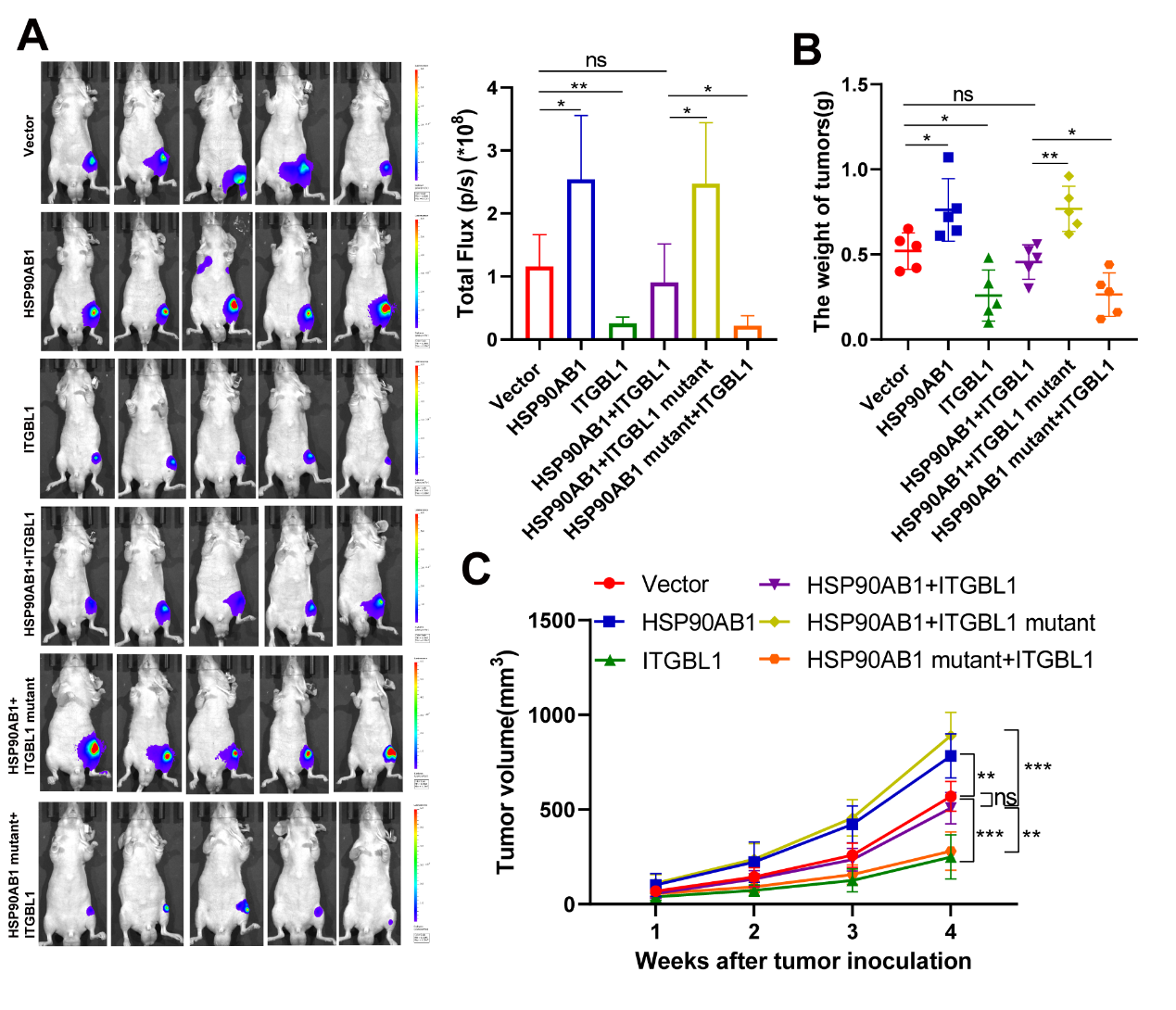


**Figure S9. Effects of HSP90AB1-ITGBL1 axis on OS growth *in vivo*. A**. Bioluminescence images and quantification in orthotopic OS models (n = 5 per group). **B**. The tumor weight and **C.** growth in orthotopic OS models. All data are presented as the means ± SD, and differences between groups were assessed by student’s t test. *, *p* < 0.05; **, *p* < 0.01; ***, *p* < 0.001; ****, *p* < 0.0001.


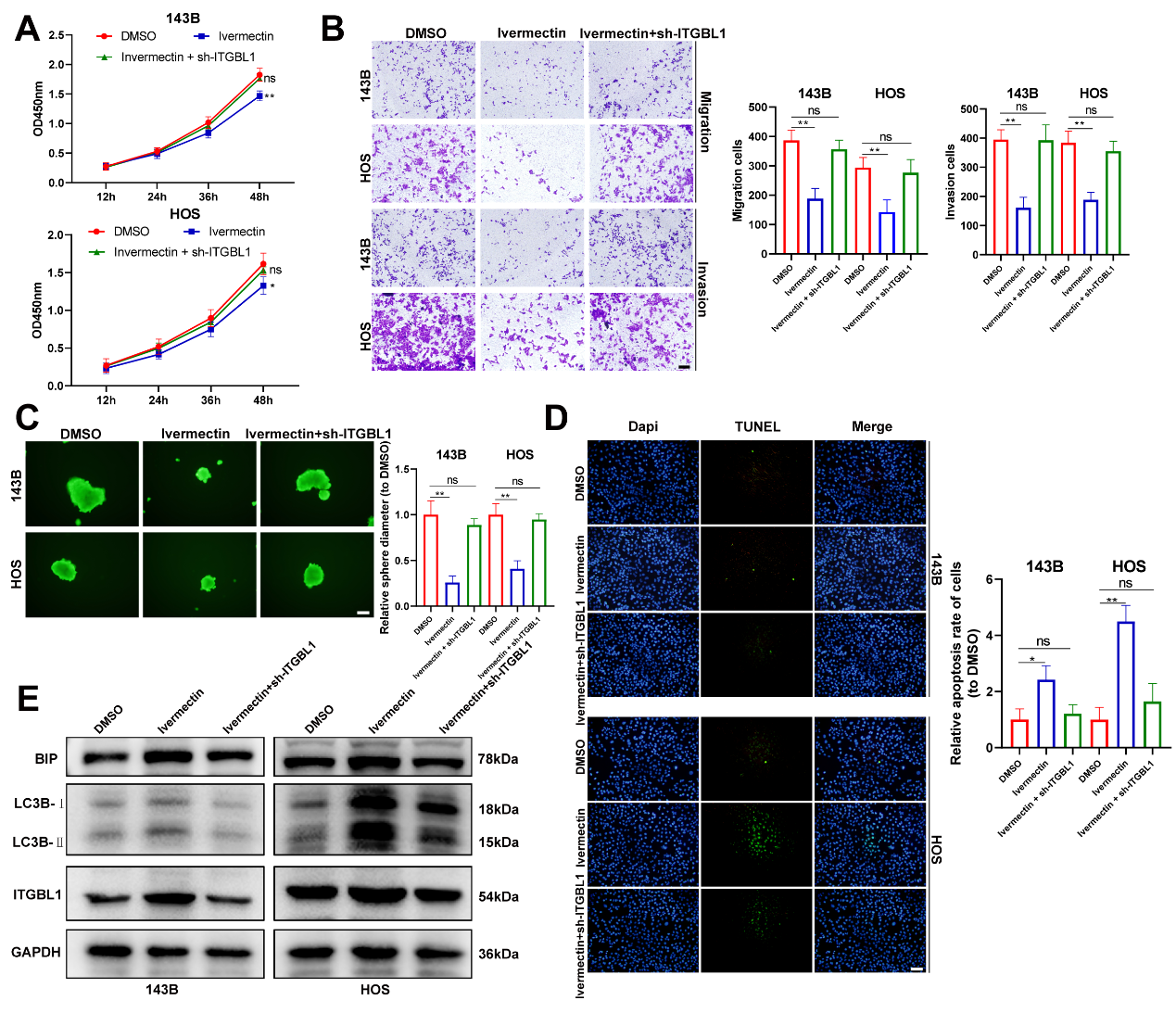


**Figure S10. Knockdown of ITGBL1 attenuates the inhibitory effects of Ivermectin on OS progression *in vitro*. A**. CCK8 assay show that knockdown of ITGBL1 weakened the inhibitory effect of ivermectin on OS cell growth. **B**. Transwell assay show that knockdown of ITGBL1 weakened the inhibitory effect of ivermectin on OS cell migration and invasion. **C**. Knockdown of ITGBL1 attenuates the inhibitory effect of ivermectin on OS cell sphere formation. Scale bar = 50 μm. **D**. TUNEL assay show that knockdown of ITGBL1 weakened the pro-apoptotic effect of ivermectin on OS cells. Scale bar = 100 μm. **E**. Western blot analysis show that knockdown of ITGBL1 in OS cells weakened the effects of ivermectin on promoting ER stress and autophagy. All data are presented as the means ± SD, and differences between groups were assessed by student’s t test. *, *p* < 0.05; **, *p* < 0.01; ***, *p* < 0.001; ****, *p* < 0.0001.
