# Supplementary material for: HSP90AB1‐Mediated Ubiquitin‐Proteasome Degradation of ITGBL1 Promotes Osteosarcoma Progression by Inhibiting Endoplasmic Reticulum Stress‐Induced Autophagy
Source: Adv Sci (Weinh). 2026 Feb 16;13(23):e15651. doi: 10.1002/advs.202515651 (PMC13104077; doi:10.1002/advs.202515651)
Supplement: Supplementary file 2 — Supporting File 2: advs74384‐sup‐0002‐SuppRawData.pptx. [file ADVS-13-e15651-s002.pptx]

## Slide 1
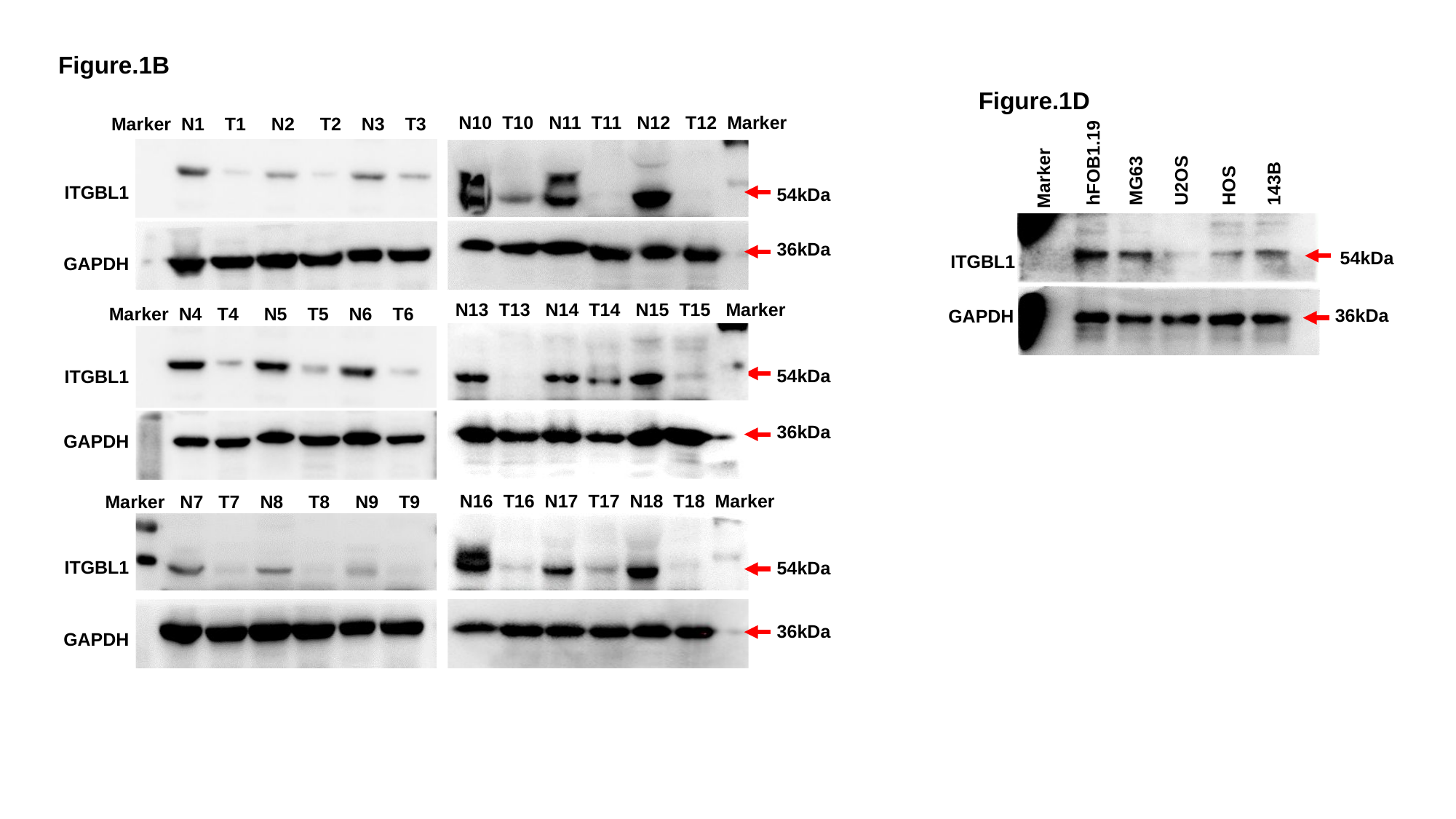

Figure.1B
Figure.1D
 N10 T10 N11 T11 N12 T12 Marker
Marker N1 T1 N2 T2 N3 T3
ITGBL1
54kDa
36kDa
GAPDH
 N13 T13 N14 T14 N15 T15 Marker
Marker N4 T4 N5 T5 N6 T6
54kDa
ITGBL1
36kDa
GAPDH
 N16 T16 N17 T17 N18 T18 Marker
Marker N7 T7 N8 T8 N9 T9
ITGBL1
54kDa
36kDa
GAPDH
hFOB1.19
U2OS
143B
MG63
Marker
HOS
54kDa
ITGBL1
36kDa
GAPDH

## Slide 2
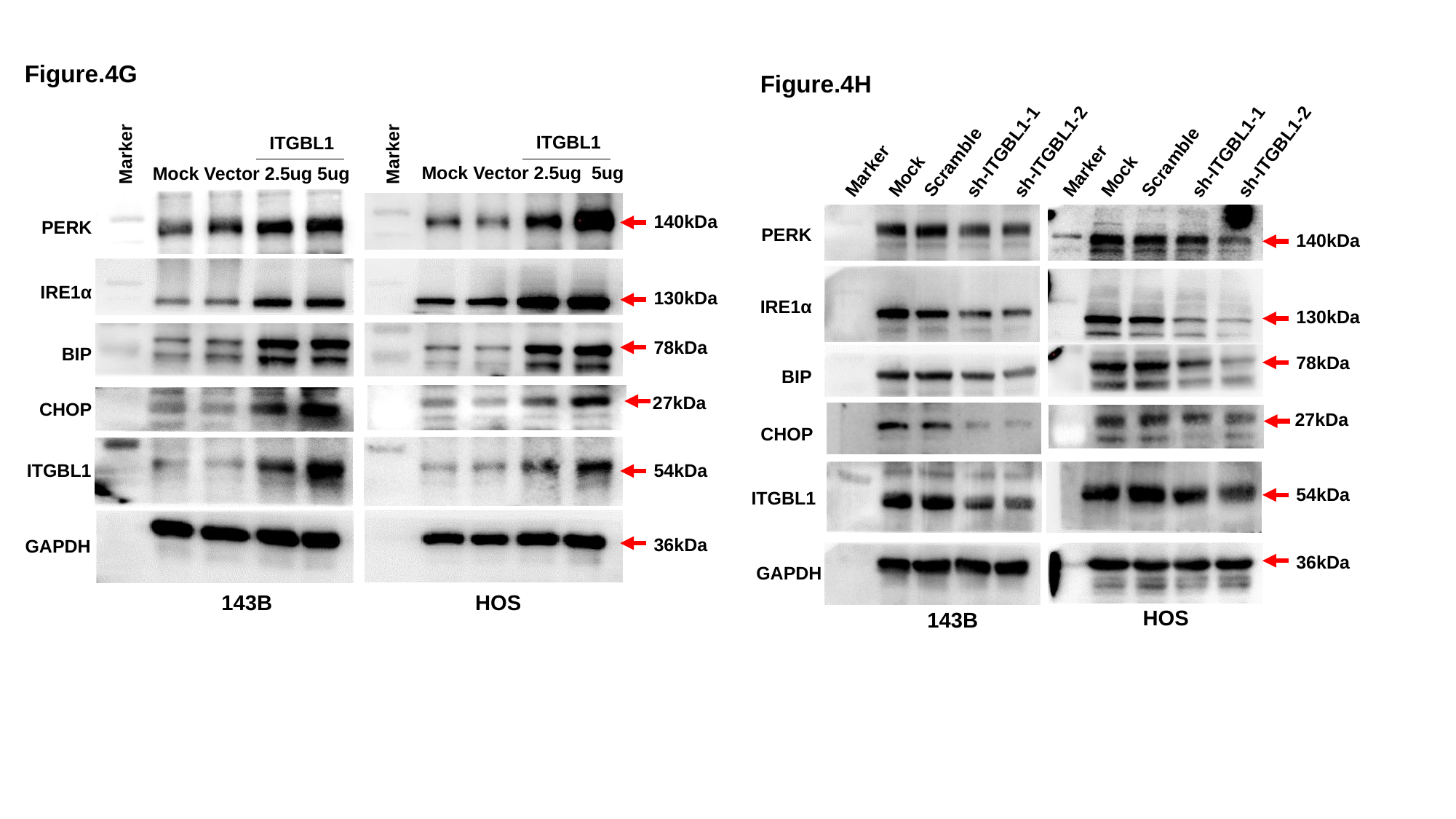

Figure.4G
ITGBL1
ITGBL1
 Mock Vector 2.5ug 5ug
 Mock Vector 2.5ug 5ug
PERK
IRE1α
BIP
CHOP
ITGBL1
GAPDH
143B
HOS
Marker
Marker
27kDa
54kDa
Figure.4H
sh-ITGBL1-2
sh-ITGBL1-2
sh-ITGBL1-1
sh-ITGBL1-1
Scramble
Scramble
Marker
Mock
Marker
Mock
PERK
140kDa
IRE1α
130kDa
78kDa
BIP
27kDa
CHOP
54kDa
ITGBL1
36kDa
GAPDH
HOS
143B
140kDa
130kDa
78kDa
36kDa

## Slide 3
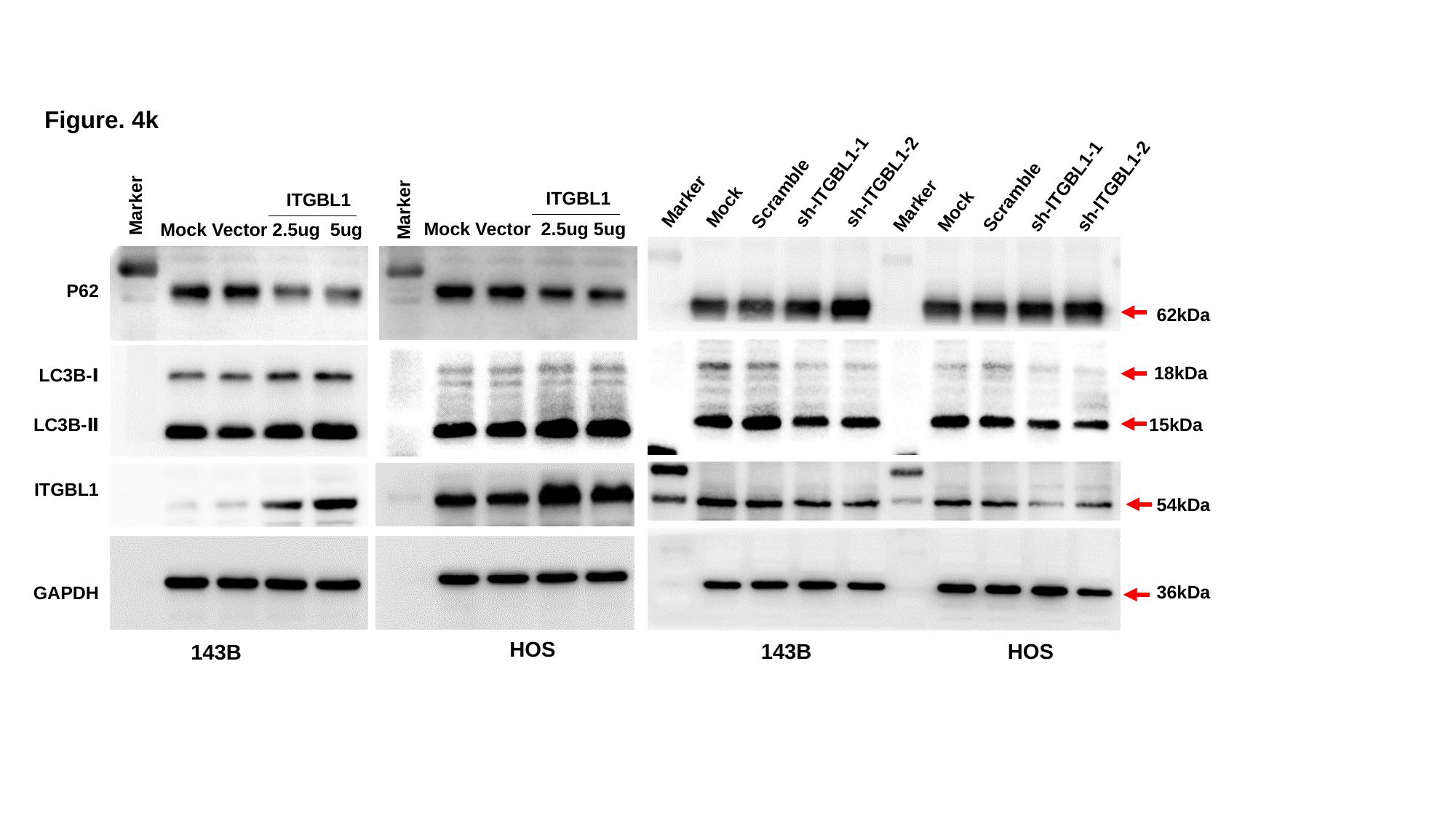

Figure. 4k
sh-ITGBL1-2
sh-ITGBL1-2
sh-ITGBL1-1
sh-ITGBL1-1
Scramble
Scramble
ITGBL1
Marker
Mock
ITGBL1
Marker
Mock
Marker
Marker
 Mock Vector 2.5ug 5ug
 Mock Vector 2.5ug 5ug
P62
62kDa
18kDa
LC3B-Ⅰ
15kDa
LC3B-Ⅱ
ITGBL1
54kDa
36kDa
GAPDH
HOS
143B
HOS
143B

## Slide 4
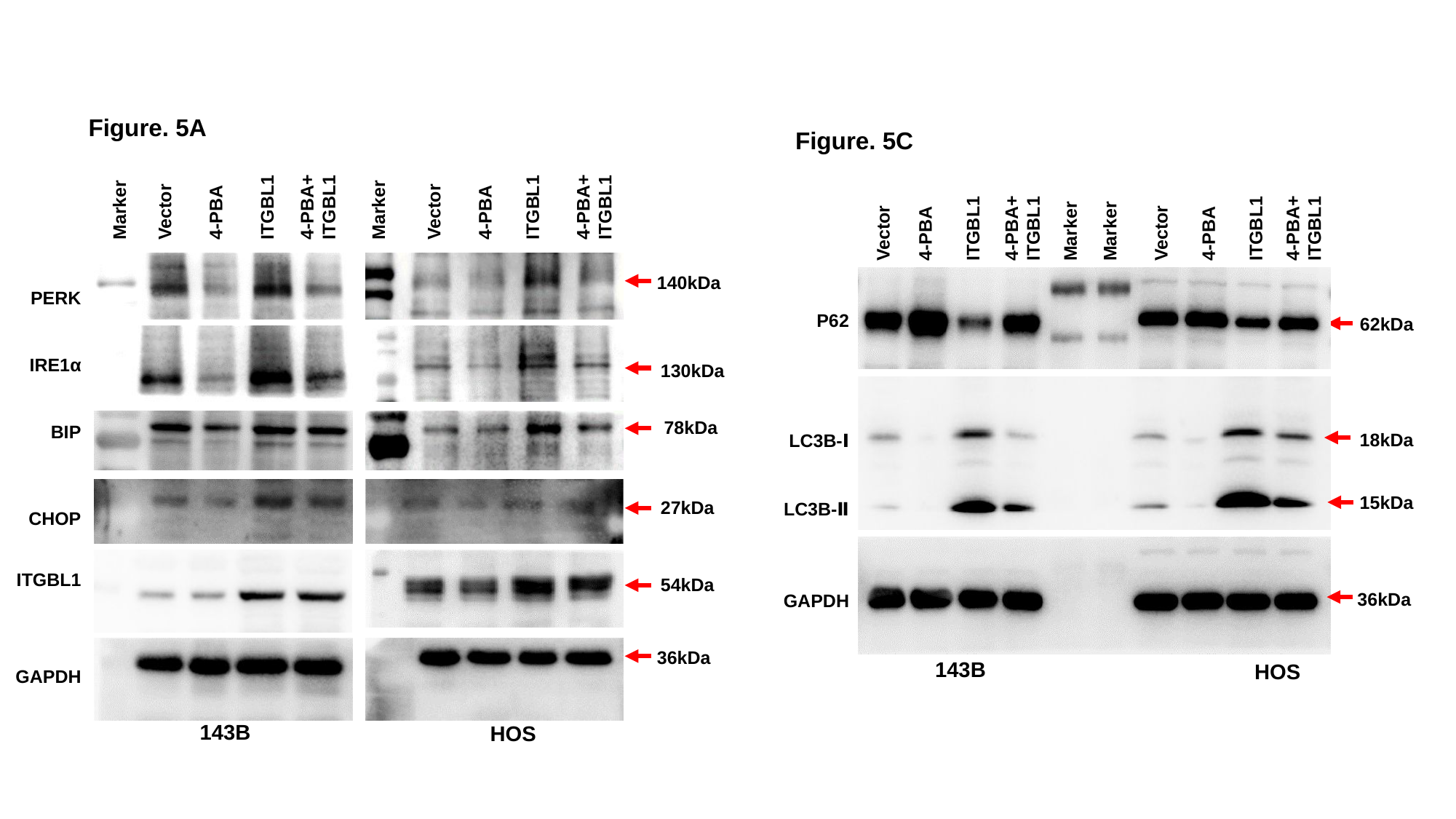

Figure. 5A
Figure. 5C
Vector
Vector
4-PBA+
ITGBL1
4-PBA+
ITGBL1
Marker
Marker
ITGBL1
ITGBL1
4-PBA
Vector
Vector
4-PBA
P62
62kDa
18kDa
LC3B-Ⅰ
15kDa
LC3B-Ⅱ
36kDa
GAPDH
143B
HOS
4-PBA
4-PBA
4-PBA+
ITGBL1
4-PBA+
ITGBL1
Marker
Marker
ITGBL1
ITGBL1
140kDa
PERK
IRE1α
130kDa
78kDa
BIP
27kDa
CHOP
ITGBL1
54kDa
36kDa
GAPDH
143B
HOS

## Slide 5
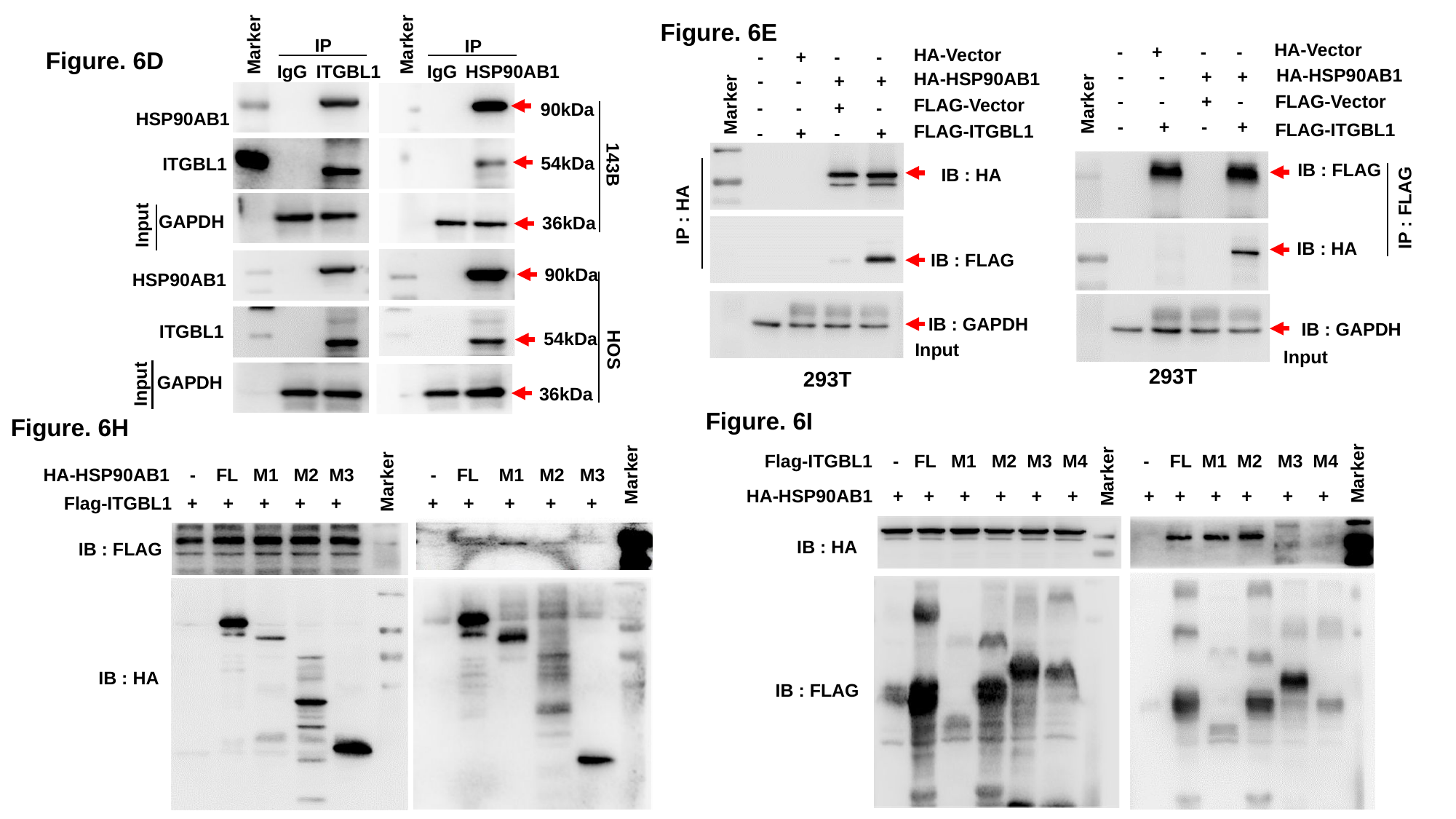

IP
IP
Figure. 6D
IgG
ITGBL1
IgG
HSP90AB1
90kDa
HSP90AB1
54kDa
ITGBL1
143B
Input
GAPDH
36kDa
90kDa
HSP90AB1
ITGBL1
54kDa
HOS
Input
GAPDH
36kDa
Marker
Marker
Figure. 6E
HA-Vector
-
+
-
-
HA-Vector
-
+
-
-
HA-HSP90AB1
-
-
+
+
HA-HSP90AB1
-
-
+
+
Marker
Marker
-
-
+
-
FLAG-Vector
FLAG-Vector
-
-
+
-
-
+
-
+
FLAG-ITGBL1
FLAG-ITGBL1
-
+
-
+
IB : FLAG
IB : HA
IP : HA
IP : FLAG
IB : HA
IB : FLAG
IB : GAPDH
IB : GAPDH
Input
Input
293T
293T
Figure. 6I
Figure. 6H
Marker
HA-HSP90AB1 - FL M1 M2 M3 - FL M1 M2 M3
Marker
Flag-ITGBL1 + + + + + + + + + +
IB : FLAG
IB : HA
Flag-ITGBL1 - FL M1 M2 M3 M4 - FL M1 M2 M3 M4
Marker
Marker
HA-HSP90AB1 + + + + + + + + + + + +
IB : HA
IB : FLAG

## Slide 6
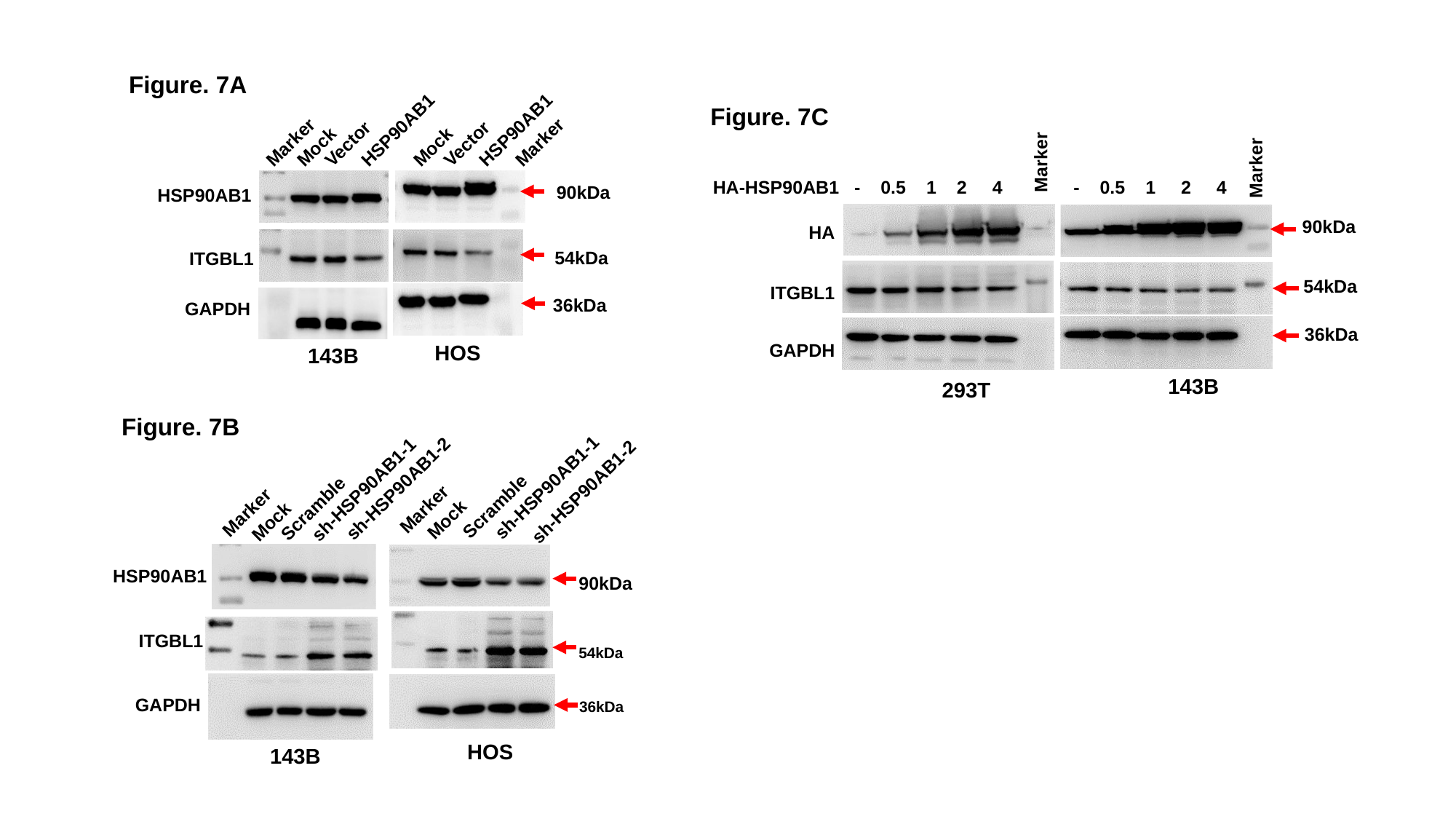

Figure. 7A
HSP90AB1
HSP90AB1
Marker
Mock
Marker
Mock
Vector
Vector
90kDa
HSP90AB1
54kDa
ITGBL1
36kDa
GAPDH
HOS
143B
Figure. 7C
HA-HSP90AB1 - 0.5 1 2 4 - 0.5 1 2 4
90kDa
HA
54kDa
ITGBL1
36kDa
GAPDH
143B
293T
Marker
Marker
Figure. 7B
sh-HSP90AB1-1
sh-HSP90AB1-2
sh-HSP90AB1-2
sh-HSP90AB1-1
Scramble
Marker
Marker
Scramble
Mock
Mock
HSP90AB1
90kDa
ITGBL1
54kDa
GAPDH
36kDa
HOS
143B

## Slide 7
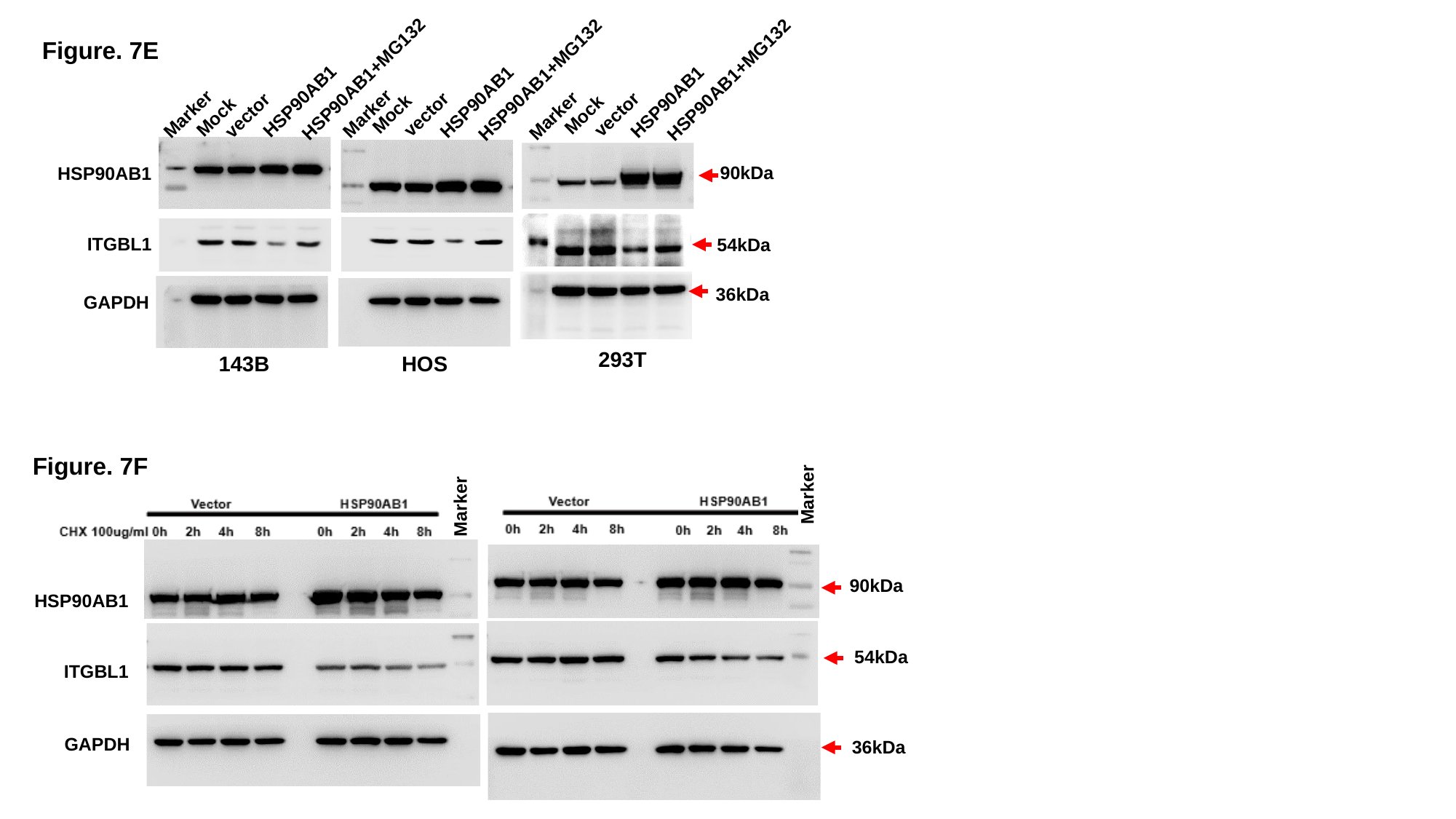

Figure. 7E
HSP90AB1+MG132
HSP90AB1+MG132
HSP90AB1+MG132
Marker
HSP90AB1
HSP90AB1
HSP90AB1
vector
vector
Marker
vector
Mock
Marker
Mock
Mock
90kDa
HSP90AB1
ITGBL1
54kDa
36kDa
GAPDH
293T
HOS
143B
Figure. 7F
Marker
Marker
90kDa
HSP90AB1
54kDa
ITGBL1
GAPDH
36kDa

## Slide 8
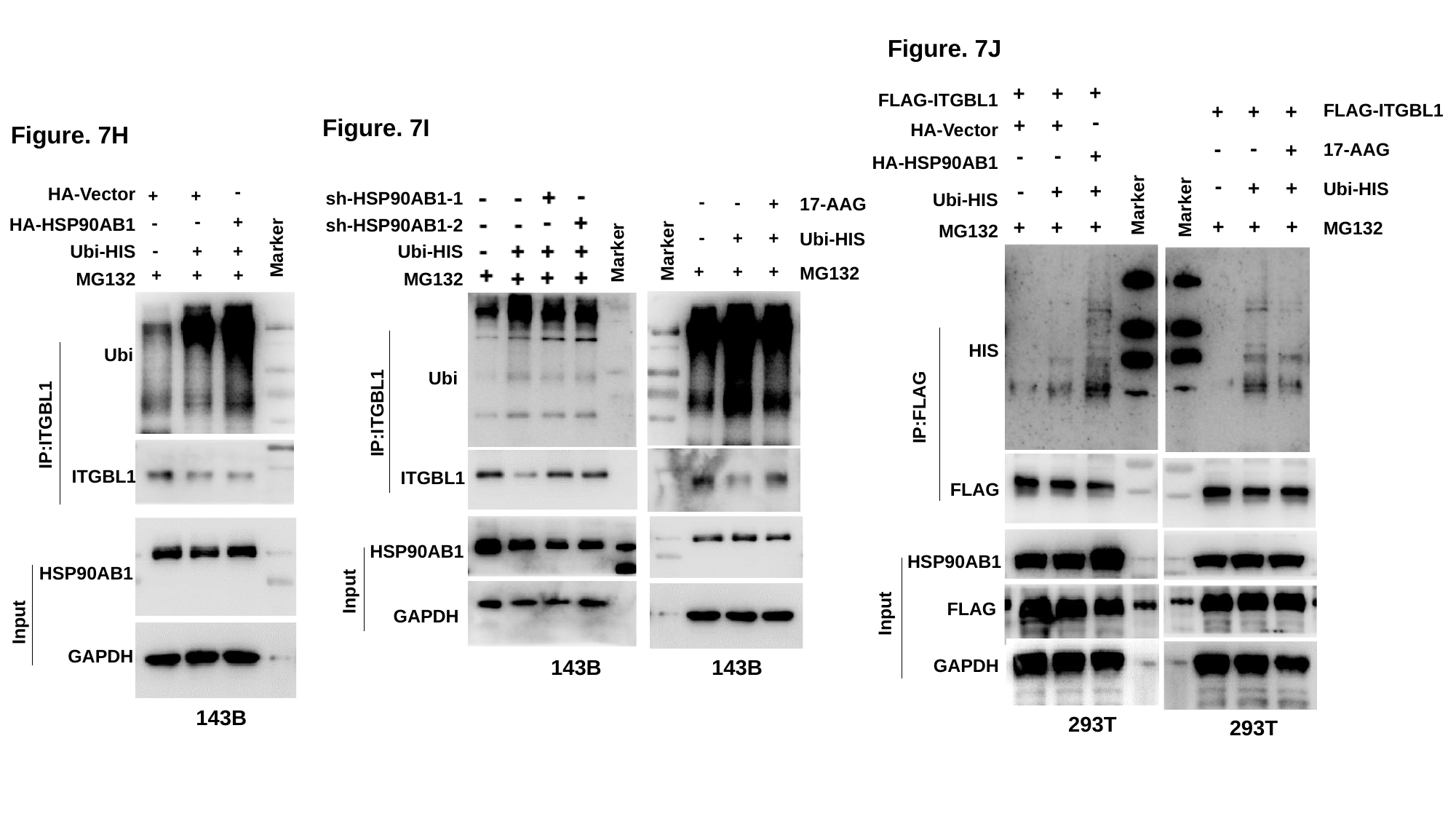

Figure. 7J
+
+
+
FLAG-ITGBL1
+
+
+
FLAG-ITGBL1
-
+
+
HA-Vector
-
-
+
17-AAG
-
-
+
HA-HSP90AB1
-
+
+
Ubi-HIS
-
+
+
Marker
Marker
Ubi-HIS
+
+
+
+
+
+
MG132
MG132
HIS
IP:FLAG
FLAG
HSP90AB1
Input
FLAG
GAPDH
293T
293T
Figure. 7I
sh-HSP90AB1-1
-
-
+
17-AAG
sh-HSP90AB1-2
-
+
+
Ubi-HIS
Marker
Marker
Ubi-HIS
+
+
+
MG132
MG132
Ubi
IP:ITGBL1
ITGBL1
HSP90AB1
Input
GAPDH
143B
143B
Figure. 7H
-
HA-Vector
+
+
-
+
-
HA-HSP90AB1
Marker
-
Ubi-HIS
+
+
+
+
+
MG132
Ubi
IP:ITGBL1
ITGBL1
HSP90AB1
Input
GAPDH
143B

## Slide 9
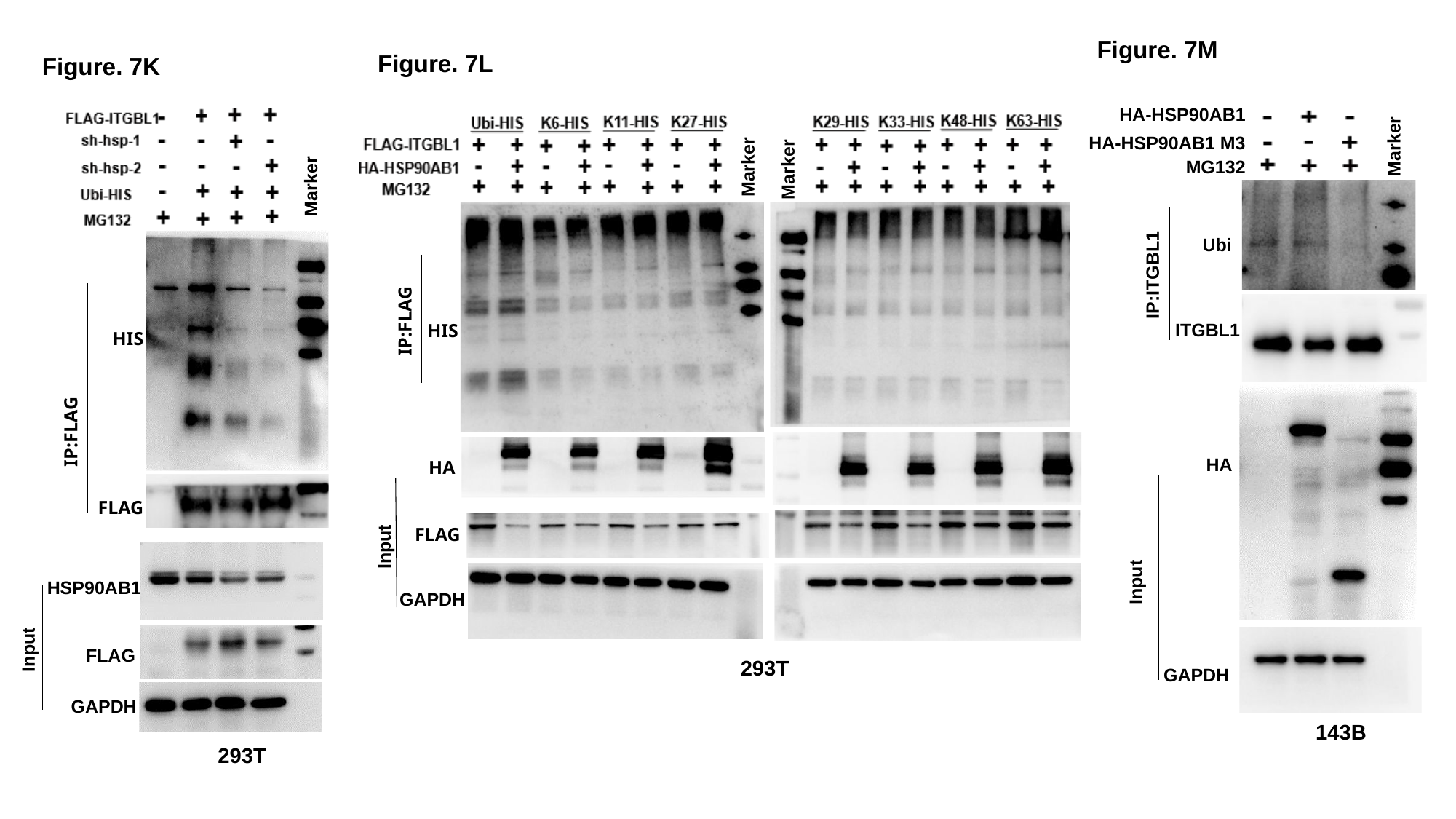

Figure. 7M
HA-HSP90AB1
Marker
HA-HSP90AB1 M3
MG132
Ubi
IP:ITGBL1
ITGBL1
HA
Input
GAPDH
143B
Figure. 7L
Marker
Marker
IP:FLAG
HIS
HA
FLAG
Input
GAPDH
293T
Figure. 7K
Marker
HIS
IP:FLAG
FLAG
HSP90AB1
Input
FLAG
GAPDH
293T

## Slide 10
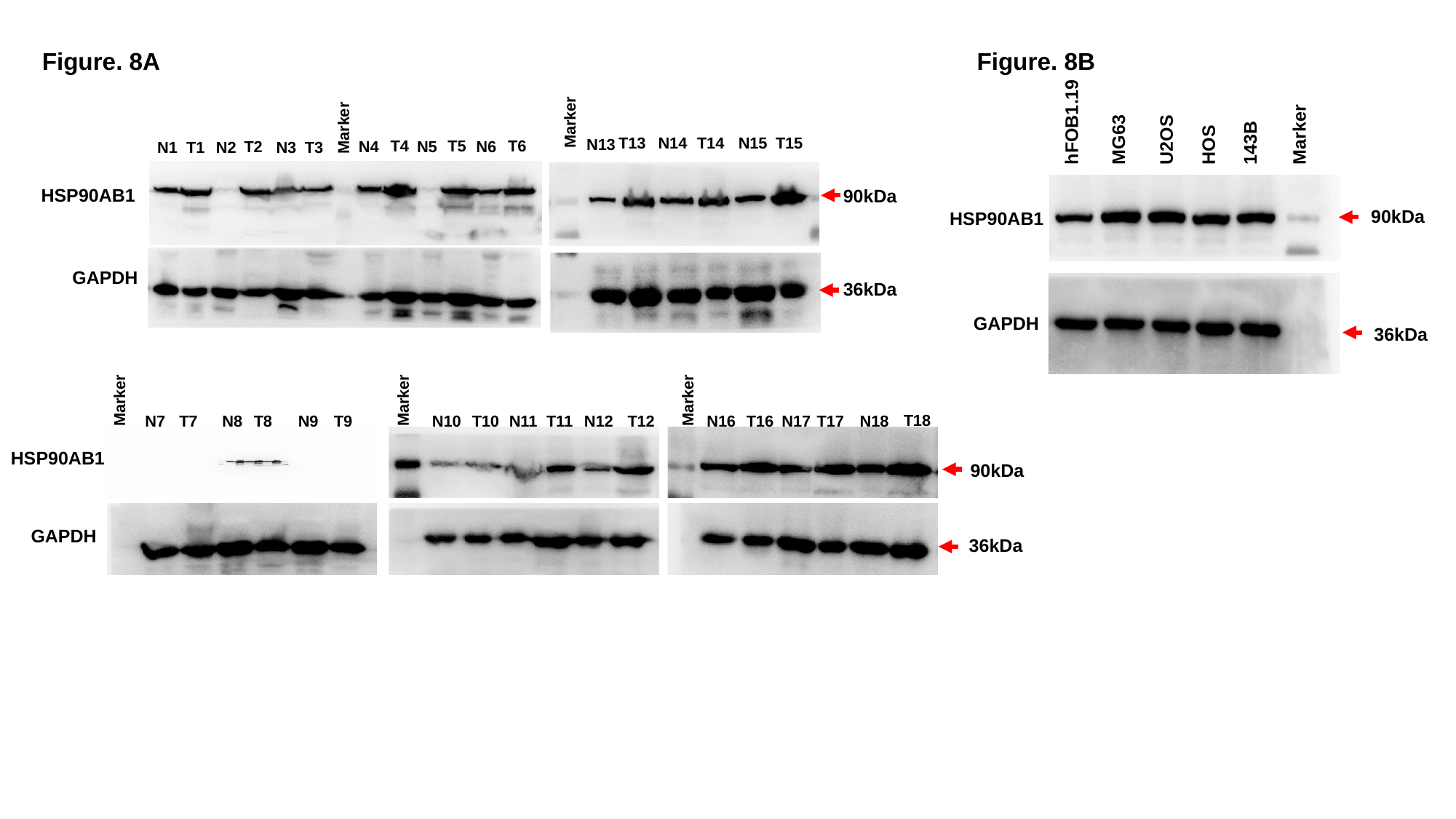

Figure. 8A
Figure. 8B
hFOB1.19
U2OS
143B
MG63
Marker
HOS
90kDa
HSP90AB1
GAPDH
36kDa
Marker
Marker
T13
N14
T14
N15
T15
N13
T5
T6
T4
N6
N5
N4
T2
T1
N3
N2
N1
T3
HSP90AB1
90kDa
GAPDH
36kDa
Marker
Marker
Marker
T18
N7
T7
N8
T8
N9
T9
N10
T10
N11
T11
N12
T12
N16
T16
N17
T17
N18
HSP90AB1
90kDa
GAPDH
36kDa

## Slide 11
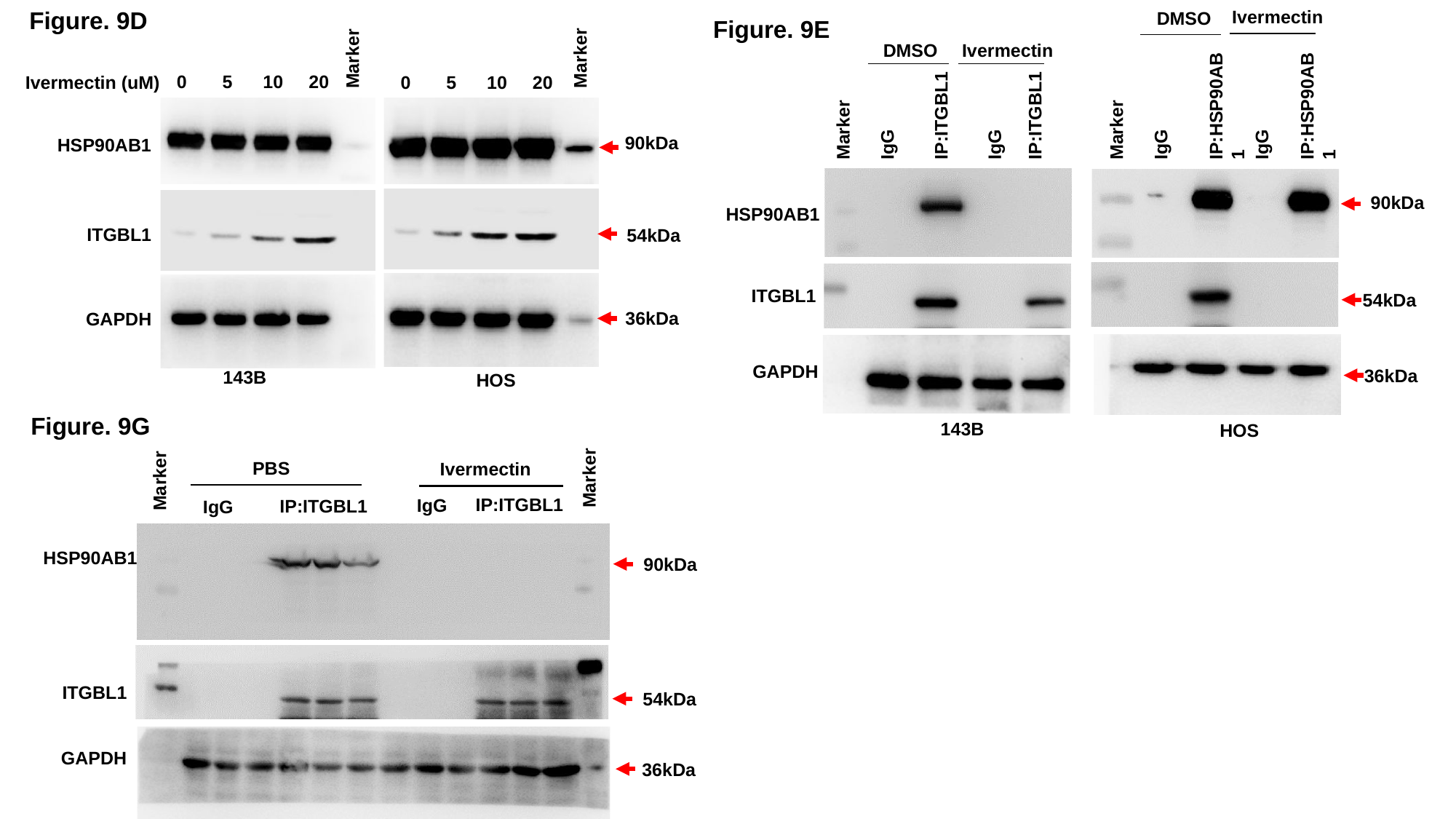

Figure. 9D
Marker
Marker
 0 5 10 20
 0 5 10 20
Ivermectin (uM)
90kDa
HSP90AB1
ITGBL1
54kDa
36kDa
GAPDH
143B
HOS
Ivermectin
DMSO
Figure. 9E
Ivermectin
DMSO
IP:ITGBL1
IP:HSP90AB1
IP:HSP90AB1
IP:ITGBL1
IgG
IgG
Marker
Marker
IgG
IgG
90kDa
HSP90AB1
ITGBL1
54kDa
GAPDH
36kDa
143B
HOS
Figure. 9G
PBS
Ivermectin
Marker
Marker
IP:ITGBL1
IgG
IP:ITGBL1
IgG
HSP90AB1
90kDa
ITGBL1
54kDa
GAPDH
36kDa

## Slide 12
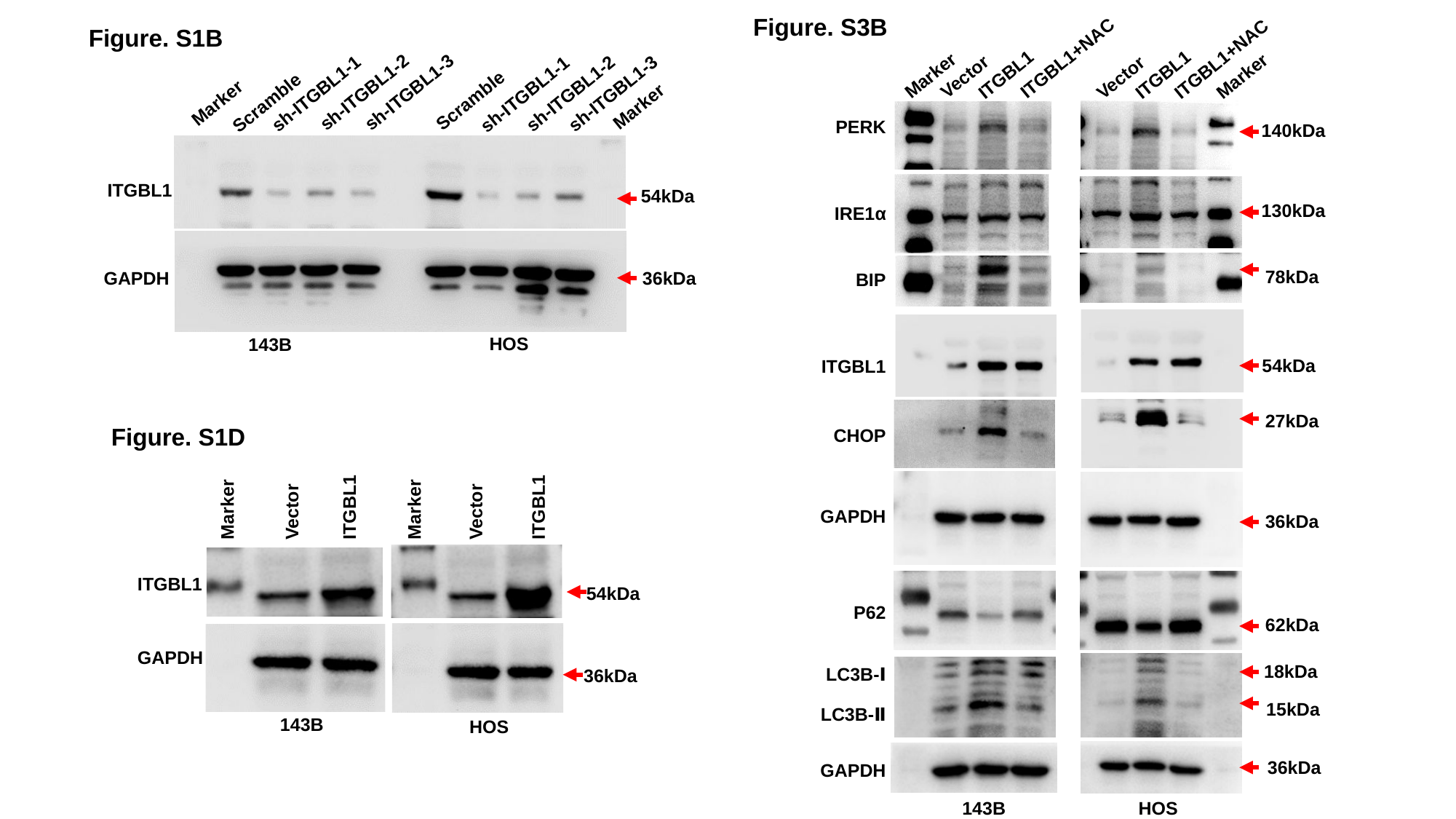

Figure. S3B
ITGBL1+NAC
ITGBL1+NAC
Vector
Marker
Marker
ITGBL1
Vector
ITGBL1
PERK
140kDa
130kDa
IRE1α
78kDa
BIP
54kDa
ITGBL1
27kDa
CHOP
GAPDH
36kDa
P62
62kDa
18kDa
LC3B-Ⅰ
15kDa
LC3B-Ⅱ
36kDa
GAPDH
HOS
143B
Figure. S1B
sh-ITGBL1-3
sh-ITGBL1-3
Scramble
sh-ITGBL1-2
sh-ITGBL1-1
sh-ITGBL1-2
sh-ITGBL1-1
Scramble
Marker
Marker
ITGBL1
54kDa
GAPDH
36kDa
HOS
143B
Figure. S1D
Vector
ITGBL1
Vector
ITGBL1
Marker
Marker
ITGBL1
54kDa
GAPDH
36kDa
143B
HOS

## Slide 13
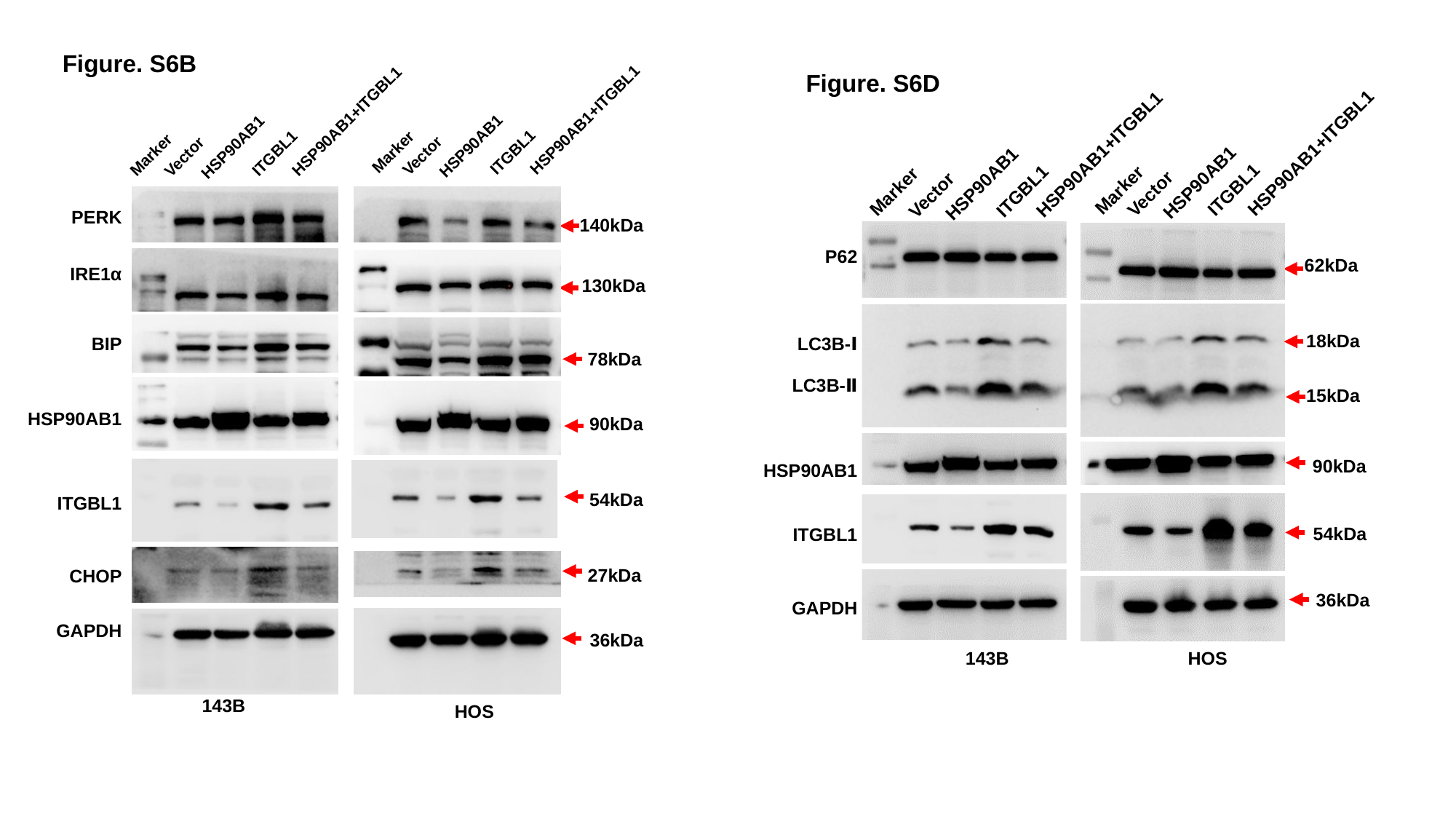

Figure. S6B
HSP90AB1+ITGBL1
HSP90AB1+ITGBL1
HSP90AB1
HSP90AB1
Marker
ITGBL1
Marker
ITGBL1
Vector
Vector
PERK
140kDa
IRE1α
130kDa
BIP
78kDa
HSP90AB1
90kDa
54kDa
ITGBL1
27kDa
CHOP
GAPDH
36kDa
143B
HOS
Figure. S6D
HSP90AB1+ITGBL1
HSP90AB1+ITGBL1
HSP90AB1
HSP90AB1
Marker
Marker
ITGBL1
ITGBL1
Vector
Vector
P62
62kDa
18kDa
LC3B-Ⅰ
LC3B-Ⅱ
15kDa
90kDa
HSP90AB1
54kDa
ITGBL1
36kDa
GAPDH
143B
HOS

## Slide 14
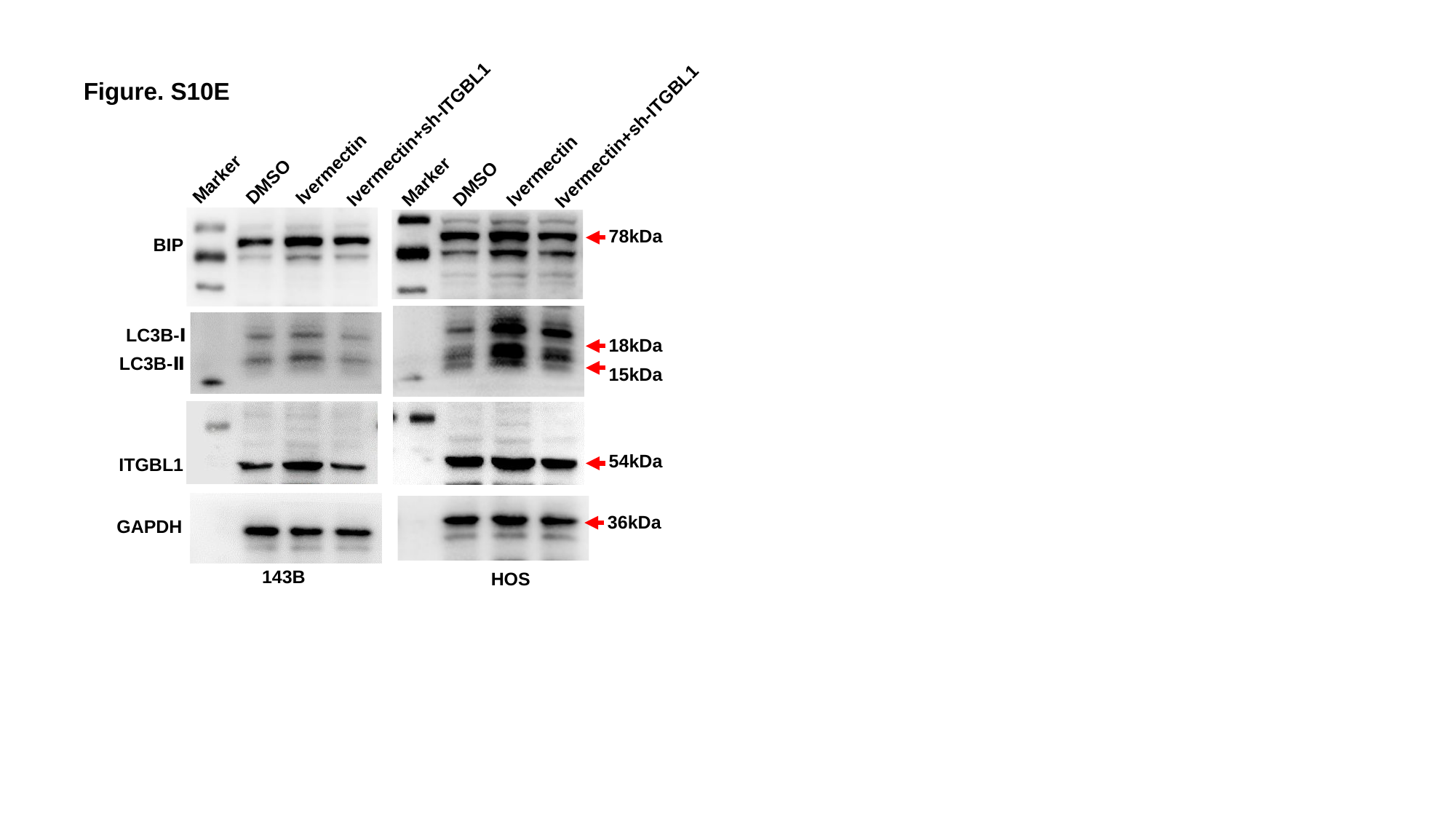

Figure. S10E
Ivermectin+sh-ITGBL1
Ivermectin+sh-ITGBL1
Ivermectin
Ivermectin
Marker
Marker
DMSO
DMSO
78kDa
BIP
LC3B-Ⅰ
18kDa
LC3B-Ⅱ
15kDa
54kDa
ITGBL1
36kDa
GAPDH
143B
HOS
